# Spatially Optimised Approach for Predicting Water Quality in a Heterogeneous Agricultural Watershed

**DOI:** 10.1007/s10666-025-10045-x

**Published:** 2025-06-23

**Authors:** Maziar Mohammadi, Fahimeh Mirchooli, Ciriaco McMackin, Saeed Aghel, Markus Egli

**Affiliations:** 1 Department of Geography, University of Zurich, Winterthurerstrasse 190, 8057 Zurich, Switzerland; 2 Department of Geography, University of Bonn, Meckenheimer Allee 16, Bonn, 53115 Germany; 3 Department of Environmental Science, Faculty of Natural Resources, Tarbiat Modares University, Noor, 4641776489 Mazandaran Province Iran

**Keywords:** Water quality modelling, Spatially adaptive models, Machine learning algorithms, Water quality management, Watershed management

## Abstract

Predicting water quality in a heterogeneous watershed is challenging because parameters and prediction accuracy vary with space. Therefore, spatially adaptive machine learning models were introduced for predicting water quality conditions in the Haraz and Babolroud watersheds, Iran. Initially, the Irrigated Water Quality Index (IWQI) was calculated. Then, spatial clusters of 16 water quality stations having similar physiochemical characteristics were identified. In the next step, numerical prediction models were developed for each cluster by assessing the prediction accuracy of six machine learning models including support vector machine (SVM), random forest (RF), extra trees (ET), extreme gradient boosting (XGBoost), decision trees (DT), and boosted regression trees (BRT). Finally, a sensitivity analysis was carried out to investigate the sets of key parameters needed to enhance water quality prediction using locally optimised prediction models. The findings indicated that water quality varied across the study area and three clusters, based on physico-chemical characteristics of the water quality, of the monitored stations were identified. The XGBoost model gave the highest accuracy and performance in cluster 1, 2, and 3 with *R*2 values of 0.99 and RMSE values of 0.02, 0.05, and 0.02, respectively. The results indicated that acceptable local prediction can be obtained using different water quality parameters in the clusters across the watershed. Our findings can help managers and policymakers providing prompt alerts regarding irrigation water quality concerns in adaptive agricultural development.

## Introduction

Surface water is a vital resource, but its quality is constantly degrading with increasing pollution pressures from industrialization, urbanization and agriculture [1, 2]. In addition, climate change may influence water quality and contribute to a larger variability within watersheds [3, 4]. Water quality deterioration may cause environmental problems such as aquatic ecosystem destruction or human waterborne disease. As a result, continuous monitoring and prediction of water quality parameters is an indispensable part of water resource management and development strategies. To evaluate the level of water pollution, responsible agencies adopt sampling strategies (daily, weekly, or monthly) at water quality stations throughout the river using physical, chemical, and biological parameters [5]. Although monitoring is a practical method, it has several inherent challenges, including its costliness, complexity and resource intensiveness [6]. Traditional approaches of river water quality modelling are mostly based on the advection dispersion equation incorporating geochemical and hyporheic exchange processes at varying degrees of complexity [7, 8]. Alternative methods take the catchment perspective by considering the river corridor as an integral component of a multifaceted natural system [9, 10].

With the emergence of the big data era, ML models stemming from artificial intelligence have faced a revolutionary development, offering innovative methods for modelling water quality [2]. Such a development can also be attributed to the vast amount of available hydrometric data due to the improved monitoring techniques (Chen et al. 2024); [11]. ML models operate based on data-driven principles and are independent of the specific hydrological processes occurring within watersheds [12]. These models utilise algorithms to analyse the relationship between input and output data and uncovering patterns relevant to the prediction of targeted environmental phenomena [13, 14]. The key advantage of the ML-based method over traditional approaches is its focus on using the fewest possible parameters while achieving optimal accuracy [15]. Currently, four types of ML models are used including the kernel-based models [16, 17], artificial neural networks [18, 19], tree-based models [20, 21] and gradient boosting models [20, 22], which are successfully applied for water quality modelling studies. These ML models have been used to investigate water quality prediction in deep agricultural wells [23], industrially polluted streams [24] and groundwater quality [25, 26]. Performing sensitivity analyses on models is a pivotal step following their setup, offering valuable insights into managing sudden shifts in water quality across various locations. Sensitivity analyses delve into the discerning effects of parameters on water quality by evaluating their relative importance. By systematically reducing parameters, this method allows for a meticulous exploration of the acceptable accuracy in predicting water quality [27].

Recently, machine learning (ML) has greatly improved the modelling of water quality parameters, especially when prior knowledge is limited [28–31]. However, developing a global prediction model to effectively capture the diverse nature of water quality characteristics across multiple stations continues to be a challenge [32]. This difficulty arises from the spatial heterogeneity inherent in water quality data collected at different locations. Despite utilising data from all stations to construct a model, it may still show an inadequate performance with an imbalanced ability to forecast water quality across the different parts of watersheds. This means that the model may perform well for some stations but poorly for others. In other words, using data from multiple monitoring stations in a single ML model might lead to a poor prediction performance. Because the model would have to account for the complexity and variability of water quality data across various stations, it will be difficult to identify accurate patterns and relationships.

To tackle this challenge and build high-performance prediction models, an adaptive regionalisation method is necessary. This involves a division of the study area into regions based on their unique characteristics and then a local water quality prediction modelling within each region. By doing so, the model can better capture the specific variations of water quality in each region, leading to more accurate predictions [32]. Often, there is a scarcity of information about spatially adaptive strategies for the development of locally optimal water quality prediction models.

In Iran, the agricultural sector dominates water consumption, accounting for over 90% of the country’s total water withdrawals [33, 34]. The Mazandaran province, located in northern Iran, plays an important role in agricultural production, primarily driven by rice cultivation. A large proportion of this region is covered by irrigated paddy lands, croplands and orchards, mainly concentrated in the Amol-Babol plain. Positioned at the outlet point of the Haraz and Babolroud watersheds, this plain benefits from fertile soils, expansive flat terrain and available surface and groundwater resources, providing appropriate conditions. As a result, the role of river water quality management in this region cannot be overstated in ensuring sustainable agricultural production, food security and aquatic ecosystem protection. Despite the critical role of the Haraz and Babolroud rivers in supplying water resources for agriculture in the Amol-Babol Plain, only very limited research on water quality exists. For example: Mohseni-Bandpei and Yousefi [35] evaluated water quality parameters in spring and winter seasons at eight sampling stations throughout the mainstream. Their results revealed a high turbidity level in the middle and lower reaches of the river, while parameters such as BOD and faecal coliform concentrations were notably higher during the dry season. Larijani et al. [36] in employed their study about the Haraz river multiple indices to evaluate its water quality, including the National Sanitation Foundation Water Quality Index (NSFWQI), the River Pollution Index (RPI), the Weighted Arithmetic Water Quality Index (WAWQI), and the DINIUS index. The study showed seasonal and spatial variations of water quality, with conditions ranging from moderate to poor and pollution levels increasing downstream due to human activities like waste discharge and agricultural runoff. Noorbakhsh et al. [37] collected water samples from the upstream, middle and downstream stations of the Siahrod, Haraz and Babolrod river over a 2-year period (2012–2013). The National Sanitation Foundation Water Quality Index (NSFWQI) was used to evaluate water quality, considering parameters such as turbidity, total solids, temperature, pH, dissolved oxygen (DO), biochemical oxygen demand (BOD), nitrate, total phosphorus, and faecal coliform levels. The highest water quality was observed at the upstream station of the Haraz River, while the poorest quality was found at the downstream station of the Siahrood River.

While studies on water quality of the Haraz River are scarce, several key points emerge from existing literature. These studies have focused on short-term periods, which may not be representative of long-term water quality conditions. Additionally, there is a lack of spatial information regarding significant parameters affecting water quality—considering the complexity of the LULC. Moreover, no water quality model has been developed having an acceptable level of accuracy for assessing water quality patterns and interactions of physiochemical parameters. Lastly, most of the previous studies have focused on drinking water quality indices and parameters, which is not the primary use of the river in the region, because irrigation has the main demand. To address these gaps, this investigation integrates irrigation water quality indices, a comprehensive dataset from 16 hydrometric stations over a long-term period (1966 to 2020) and advanced ML models to develop a spatially adaptive water quality models, exploring the spatial variability and relationships of the key influencing factors.

In addition, a spatial analysis of common cancer incidences showed that most provinces have been identified as high-risk regions of Iran. The necessity of water quality management is even more important in Mazandaran as a tourist hub [38] because this area suffers from water pollution problems owing to agricultural pressures on water resources, particularly in the Haraz and Babolroud river watersheds that are vital for irrigation and agricultural production. As a region that relies heavily on water resources for both farming and tourism, the management of water quality becomes not only a health concern but also an economic one, making it essential to address water pollution issues based on an adaptive regional approach.

Considering the aforementioned necessities for an adaptive local water quality, our main modelling objectives were as follows: 1) to cluster the water quality stations based on physiochemical and physiographical parameters, 2) to find the significant parameters influencing water quality in each region, 3) to build predictive models using ML algorithms and select water quality parameters to predict the IWQI at water quality stations, 4) to conduct a sensitivity analysis using the parameter importance of the best model, and finally 5) to evaluate the long-term adequacy of water quality for irrigation within the Haraz and Babolroud watersheds.

## Material and Methods

**Fig. 1:**
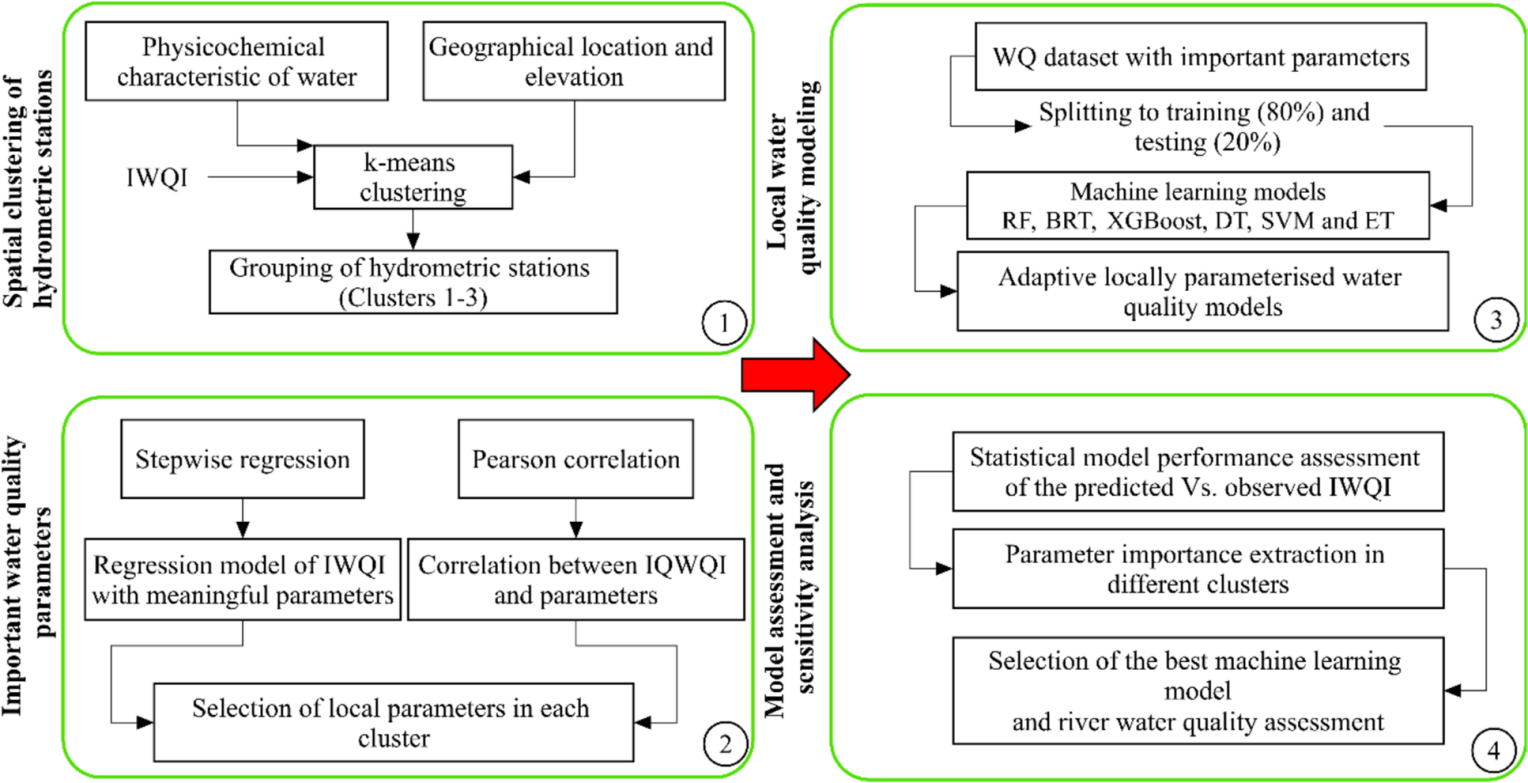
The framework of the research for enhanced water quality modelling

**Fig. 2:**
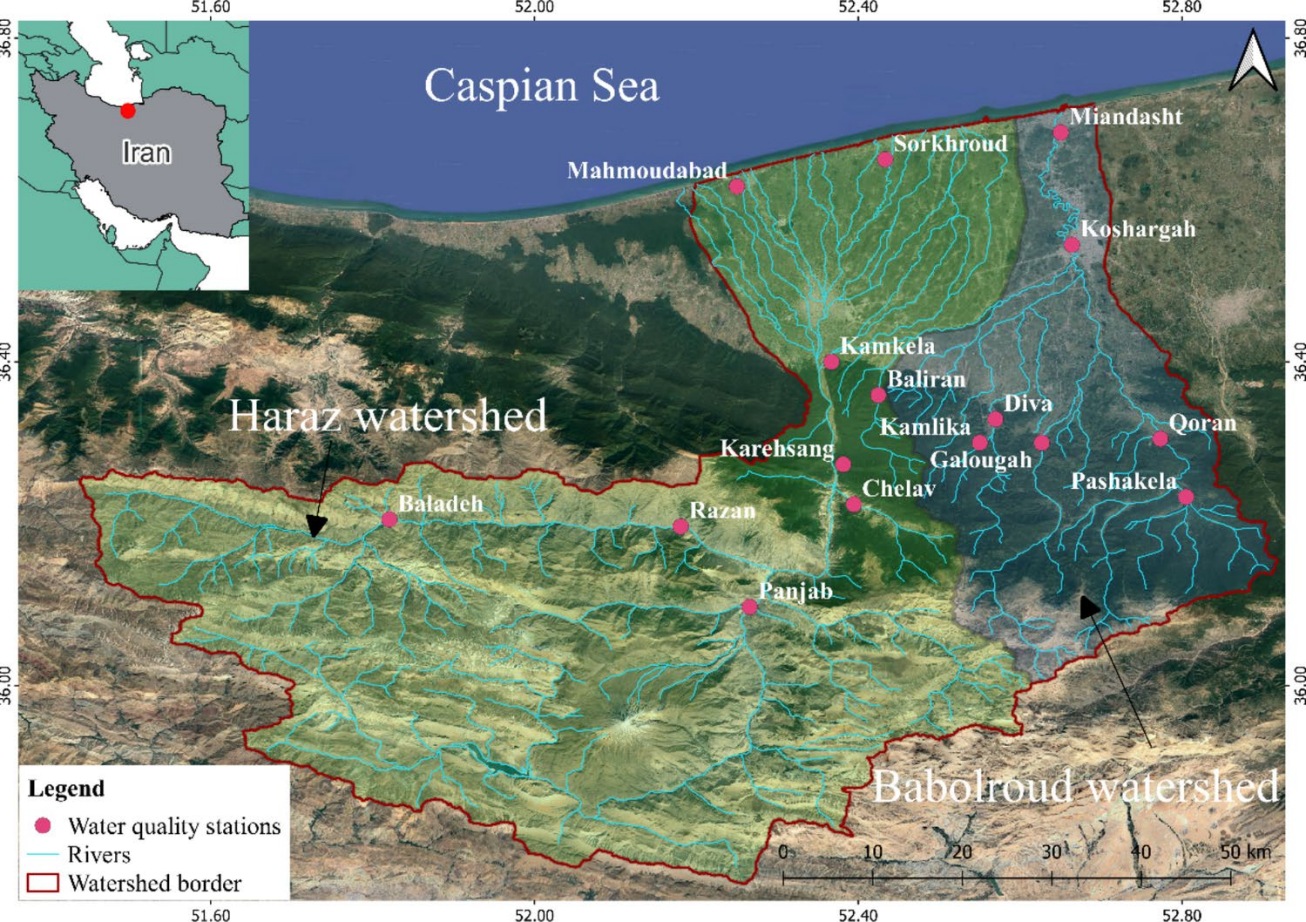
The location of the study area in northern Iran and water quality stations in the Haraz and Babolroud watersheds

In this study, six advanced tree-based ML models and the IWQI were integrated to predict water quality at hydrometric stations in two spatially heterogeneous watersheds, the Haraz and Babolroud area (Fig. 2). The overall procedure (Fig. 1) consists of four major steps: 1. processing of the datasets of the hydrometric stations, selection of water quality parameters, calculation of the IWQI and clustering the hydrometric stations, 2. regression analysis and identification of important parameters in each cluster, 3. data formatting, ML model selection and model runs using local water quality parameters, and 4. evaluation of the parameter importance and water quality assessment using irrigation water quality indices.

### Study Area

The Haraz and Babolroud watersheds are in the central region of the Mazandaran province, northern Iran, and are drained by two main rivers, namely the Haraz and Babolroud river. The study area is characterised by a mountainous area, forestland and lowlands, with elevations ranging from −10 m to 5600 m asl. and a total area of 6804 km^2^. Also, the Damavand peak, known as the highest peak of Iran, is situated in the southwest of the region. The northern part of the watershed is surrounded by a coastal strip of the Caspian Sea, while the southern part is bounded by the Central Alborz Mountains [39]. One of the main rivers of the study area, the Haraz River, originates from the Alborz Mountain, passing through Amol City, and then it flows into the Caspian Sea with braided morphology in the plain [40]. The Babolroud also originates from the Alborz Mountain but from lower elevation and reaches the Caspian Sea after passing the Babol and Babolsar cities. Mean annual rainfall is 788 mm while the highest amount occurs between November and January. The temperature of this watershed varies between 36.5 °C in summer and −25 °C (Damavand peak) in winter according to the Iranian Meteorological Department. In terms of land use, the uplands consist mostly of poor rangelands, sporadic rural and residential areas and some irrigated farming and orchards in the valleys. The middle elevations—the central part of the watersheds—are mostly covered by the Hyrcanian Forest. Finally, the plain is dominated by rice farms, orchards, cities and industrial centres (particularly the cities Amol and Babol). In addition, different sources of pollution alongside the main branch such as recreational areas, fish farming, local livestock farms, sand mines and a slaughterhouse, whose wastewater is directly and indirectly discharged into the river, influence water quality in this region (Fig. 2) [41].

### Water Quality Data

Water quality parameters can be categorised into five major groups: chemical indicators, physical indicators, bacterial indicators, biological indicators and radioactive indicators [42]. For this study, we considered Cl^−^, EC, HCO_3_^−^, TDS, pH, SO_4_^2−^, SOA, Ca^2+^, Mg^2+^, Na^+^, K^+^, SOC, Tem_H, Tot_H and SAR, which are relevant for irrigation goals. At first, the outlier detection was performed on the dataset which typically involves the calculation of the mean and standard deviation to identify values that significantly deviate from them. When outliers are identified, they can be treated either as missing values or corrected based on the context [43]. In the next step, attention was given to the missing values of the data. In this study, linear interpolation was employed to fill these gaps. Finally, data normalisation was carried out to address dimensional discrepancies among various water quality factors.

A total of 5261 monthly water quality data points were collected from 16 hydrometric stations: Haraz (Baladeh-noor, Karehsang, Razan-Noor, Panjab, Baliran, Mahmoudabad, Sorkhroud, Kamkela, Chelav) and Babolroud (Kamlika, Diva, Galougah, Koshtargah, Miandasht, Pashakola, Qaran) from 1966 to 2020. These twelve hydrometric stations are distributed across three regions: the mountainous area, the mid-elevation forested lands and the Amol-Babol plain in the vicinity of the Caspian Sea. First, the IWQI was calculated using Cl, EC, HCO_3_^−^, Na^+^ (%) and SAR. The dataset was then split into a training phase (80%) and a testing phase (20%) for predicting the IWQI at the hydrometric stations. Finally, water quality was evaluated using the remaining parameters and indices including Kelly’s ratio, magnesium hazards, percent sodium (%Na), PI, SAR, SSP, and Wilcox diagram for irrigation purposes.

### Water Quality Indices

#### IWQI

In recent decades, numerous water quality indices have been developed as practical tools to evaluate water for drinking or irrigation purposes [44–47]. A water quality index consists of various water quality parameters that are measured over time in different locations. The primary function of these indices is to simplify intricate biophysiochemical parameters into more practical and straightforward information for effective water resource management. Within this context, the IWQI stands out as a highly valuable indicator for assessing surface and groundwater resources in agricultural regions [48]. The IWQI evaluates the potential hazard related to the soil and crop triggered by water quality parameters. This index was calculated using five main parameters such as EC, SAR, Na^+^, Cl^−^ and the bicarbonate ion concentration HCO_3_^−^ [49]. To do this, the *qi* for each parameter was calculated using Eq. 1 considering the proposed *Wi* and parameter limiting range (Tables 1 and 2):

1 $$qi={q}_{max}- \frac{\left({x}_{ij}-{x}_{inf}\right)\times {q}_{iamp}}{{q}_{amp}},$$

where the ${q}_{max}$ is the upper bound of the corresponding class of $qi$, ${x}_{ij}$ indicates the measured value of the parameters shown in Table 1, ${x}_{inf}$ refers to the lower bound value of the class to which the observed parameter belongs, ${q}_{iamp}$ represents the class amplitude for *qi* classes and ${q}_{iamp}$ corresponds to class amplitude to which the parameter belongs [48]. Table 3 classifies the water quality condition regarding the IWQI.

**Table 1:** The parameters used for calculating the IWQI and proposed limiting values [49]

qi	EC (μS/cm)	SAR (meq/L)	Na^+^ (meq/L)	Cl^−^ (meq/L)	HCO_3_^−^ (meq/L)
85–100	200–750	< 3	2–3	< 4	1–1.5
60–85	750–1500	3–6	3–6	4–7	1.5–4.5
35–60	1500–3000	6–12	6–9	7–10	4.5–8.5
0–35	< 200 or > 3000	> 12	< 2 or > 9	> 10	< 1 or > 8.5

**Table 2:** The proposed weight for the parameters of the IWQI [49]

IWQI Parameters	Wi
EC	0.211
SAR	0.204
Na^+^	0.202
Cl^−^	0.194
HCO^−^	0.189
Total	1.000

**Table 3:** Water quality as derived from the IWQI [49]

WQI classes	Restriction	Recommendation	
		For soil	For plant
85 ≤ 100	No restriction (NR)	May be used for most soils with low probability of causing salinity and sodicity problems, being recommended leaching within irrigation practices, except for in soils with extremely low permeability	No toxicity risk for most plants
70 ≤ 85	Low restriction (LR)	Recommended for use in irrigated soils with light texture or moderate permeability, being recommended salt leaching. Soil sodicity in heavy texture soils may occur, being recommended to avoid its use in soils with high clay levels 2:1	Avoid salt sensitive plants
55 ≤ 70	Moderate restriction (MR)	May be used in soils with moderate to high permeability values, being suggested moderate leaching of salts	Plants with moderate tolerance to salts may be grown
40 ≤ 55	High restriction (HR)	May be used in soils with high permeability without compact layers. High frequency irrigation schedule should be adopted for water with EC above 2.00 dS m^−1^ and SAR above 7.0	Should be used for irrigation of plants with moderate to high tolerance to salts with special salinity control practices, except water with low Na, Cl and HCO_3_^−^ values
0 ≤ 40	Severe restriction (SR)	Should be avoided its use for irrigation under normal conditions. In special cases, may be used occasionally. Water with low salt levels and high SAR require gypsum application. In high saline content water soils must have high permeability, and excess water should be applied to avoid salt accumulation	Only plants with high salt tolerance, except for waters with extremely low values of Na, Cl and HCO_3_^−^

### Kelly’s Ratio (KR)

This index determines the irrigation water quality using Na, Ca and Mg concentrations (Eq. 2). KR values ≤ 1 indicate that water is suitable for irrigation and values > 1 show that the water is not appropriate [50]:

2 $$KR= \frac{{Na}^{+}}{{Ca}^{2+}+{Mg}^{2+}}.$$

### Magnesium Hazards (MHs)

The magnesium hazard [50] index is another water quality indicator to assess the suitability of water for irrigation, which is critical for crop productivity. Elevated concentrations of this ion adversely impact soil structure, leading to increased alkalinity and hinder plant growth. Based on this indicator, the water can be classified into two groups: acceptable (MHs < 50) and unacceptable (MHs > 50). This index was calculated using the following Eq. 3:

3 $$MH= \frac{{Mg}^{2+}}{{Mg}^{2+}+ {Ca}^{2+}}.$$

### Permeability Index (PI)

The permeability index introduced by Doneen [51] classifies water for irrigation into three categories: Class 1 (PI > 75%), Class 2 (25% < PI < 75%) and Class 3 (PI < 25%). Class 1 and Class 2 are designated as good and suitable, respectively, exhibiting a higher maximum permeability. The soil's permeability is influenced by the concentrations of Na^+^, Mg^2+^, Ca^2+^, and HCO_3_^−^ ions. The calculation of the PI is done as follows:

4 $$PI= \frac{Na+ \sqrt{{Hco}_{3}}}{ Ca+Mg+Na}.$$

### Percent Sodium (%Na)

The sodium percent (%Na) index was developed by [52] and classifies the water suitability into five classes such as 0 ≤ %Na ≤ 20% = excellent water, 20% < %Na ≤ 40% = good water, 40% < %Na ≤ 60% = permissible, 60% < %Na ≤ 80% = doubtful and 80% < %Na ≤ 100 = unsuitable. The percentage of the Na was determined by Eq. 5:

5 $$Na\%= \frac{{Na}^{+}+{K}^{+} }{ {Ca}^{2+}+{Mg}^{2+}+{K}^{+}+{Na}^{+}}\times 100.$$

### Sodium Adsorption Ratio (SAR)

The SAR, also referred to the sodium content or alkali hazard, is an important index to assess the suitability of water for irrigation purposes (Eq. 6). High concentrations of this element in water can lead to negative effects on soil properties, causing a reduction in soil permeability [53]. An increased salinity disrupts osmotic activities, resulting in a decreased absorption of water and nutrients from the soil. This interference disrupts the movement of water to the plant leaves and obstructs plant metabolism. The following equation given by the U.S. Department of Agriculture Salinity Laboratory in 1954 was used to calculate this indicator [52]:

6 $$Na\%= \frac{{Na}^{+} }{\sqrt{\frac{{Ca}^{2+}+{Mg}^{2+}}{2}}}.$$

### Soluble Sodium Percentage (SSP)

The soluble sodium percentage is another water quality indicator to determine water quality into unsuitable (SSP < 50%) and suitable (SSP > 50%) using Ca^+2^, Mg^+2^ and Na^+^ (Eq. 7):

7 $$\text{SSP }= \frac{Na}{Ca+Mg+Na}\times 100.$$

### Clustering of Water Quality Stations and Local Parameter Exploration

The k-means clustering was used to group the hydrometric stations using water quality parameters such as Cl, EC, HCO_3_^−^, TDS, pH, SO4, sum of anions, Ca, Mg, Na, K, sum of cations, temporary hardness, and total hardness. Furthermore, two physiographical watershed characteristic such as the spatial location (UTM) and elevation (m) were also considered for clustering the water quality stations as they have great indirect effects on the type of land use, land cover and human activity around the riparian zone [54, 55]. K-means clustering provides an effective way to group monitoring stations based on similarities in water quality parameters, facilitating the identification of spatial and temporal patterns within complex datasets [56, 57]. In this study, the Elbow method [58] was applied to select the optimal number of clusters. The results indicated that choosing three clusters (*k* = 3) minimizes the risks of both underfitting and overfitting. After clustering the water quality stations, the local parameters were explored using a stepwise regression and the Pearson correlation. Using these methods, the regression model and the import water quality parameters in IWQI were obtained and used for the modelling the related cluster with using ML models [32].

### ML Models

Six ML models were selected for this study such as the support vector machine (SVM), the random forest (RF), the extra trees (ET), the extreme gradient boosting (XGBoost), decision trees (DT), and boosted regression trees (BRT) to capture a diverse range of modelling approaches. These models represent different types of algorithms, including ensemble methods (RF, ET, XGBoost, BRT) and non-ensemble methods (SVM, DT), allowing for a robust comparison of performance. Ensemble methods are known for their high predictive accuracy and ability to handle complex datasets [59], while individual models like SVM and DT provide insights into specific relationships and feature importance [60]. By using a combination of these models, we aim to increase the reliability and generalizability of the results, ensuring that the model outcomes are not overly dependent on the characteristics of any single algorithm.

### Random Forest (RF)

RF is an ensemble ML technique introduced by [54] to address issues such as overfitting and instability associated with single decision trees. The main concept behind RF is to construct multiple decision trees independently on random subsets of the original training data. The predictions from these individual trees are averaged to enhance the model’s generalisability and robustness. Each tree in the training subset is created using a bootstrapping procedure, dividing the training dataset into an “in-bag” subset for training the decision tree and an “out-of-bag (OOB)” subset excluded from the training process. This unique partitioning for each tree enables internal validation. The OOB samples from each tree can be used to assess its performance. Averaging all OOB predictions provides an overall accuracy metric for the RF model. Typically, the in-bag and out-of-bag subsets for a decision tree are set at 66.67% and 33.33% (2:1 ratio) of the original training data, respectively. After training on the dataset, the model's performance is evaluated using a test dataset, generating OOB predictions for both the training and test sets [61].

### Boosted Regression Tree (BRT)

The BRT model combines ML and statistical methods to improve the prediction of a single model. Boosting is an adaptive approach that combines multiple regression trees to enhance predictive performance, while regression trees establish relationships between responses and influencing factors through recursive binary splits. Compared to other ML techniques, BRT offers advantages in handling various types of variables, managing missing data without the need for data transformation, and being robust to data distribution and outliers. Additionally, BRT can illustrate complex nonlinear relationships, provide high prediction accuracy and offer flexibility in modelling [1].

The performance of the BRT model is influenced by factors such as the learning rate, bag fraction, tree complexity and cross-validation as discussed in [62]. The learning rate controls the contribution of each tree to the model, while the bag fraction determines the proportion of data used in each step of model building. Tree complexity, representing the number of nodes in a tree, regulates the interactions level within the BRT model. Additionally, cross-validation helps to identify the optimal number of trees for the BRT model [63].

### XGBoost

XGBoost is a powerful regression model that uses ensemble learning techniques, including gradient boosting and decision trees, to achieve accurate predictions. XGBoost prepares various enhancements for performance while maintaining a structure like other gradient-boosting regression models [64]. The XGBoost algorithm is an enhanced version of the gradient-boosted decision tree (GDBT) algorithm. It incorporates a second-order Taylor expansion of the loss function and introduces a regularization term to prevent overfitting and accelerates convergence speed. By iteratively creating new decision trees to fit the residuals of previous predictions, XGBoost significantly enhances prediction accuracy by steadily reducing the discrepancies between predicted and actual values.

XGBoost is a cutting-edge tool for massively parallel boosting trees, currently recognised as the fastest and most advanced open-source boosting tree toolkit. Its speed surpasses common toolkits by more than 10 times [65]. It has been employed in many different fields such as in hydrology [66], remote sensing [67] and medicine [68].

### Extra Trees (ET)

The ET [69] algorithm is a tree-based ensemble learning method suitable for classification and regression tasks. In contrast to traditional tree-based approaches, ERT randomly selects attributes and split points when growing each tree [70]. Compared to the bagging-based random forest model, ERT offers two key advantages: (1) ERT incorporates all samples in tree development, enhancing model accuracy, whereas random forest relies on bagging for sample selection; (2) ERT employs random sampling and feature selection at tree nodes, leading to more effective data interpretation [71]. This random modelling approach significantly boosts predictive performance. Therefore, we used ERT for modelling and regression prediction within a comprehensive tree-based algorithm, evaluating its performance against other ML techniques.

### Support Vector Machine (SVM)

Support vector machines have been used for classification and forecasting in different studies. The latter uses the regression-based method called support vector regression (SVR). SVR can handle various types of nonlinearity mapping using a limited dataset and effectively generalises based on statistical theories. The method aims to identify the optimal linear regression for nonlinear mapping functions by employing a kernel function. It can map input data to a high-dimensional space and determine the hyperplane with the smallest distances to all data points [72].

### Decision Trees (DT)

The Decision Trees (DT) procedure was used for both regression and classification of data [73]. DT is structured with leaf nodes representing classes and non-leaf nodes containing attribute names leading to other decision trees based on attribute values. The top-down induction process of decision tree creation begins at the root node, progressing to form sub-trees until reaching leaf nodes [74]. One of the key strengths of this model is its clarity and ease of interpretation, providing a transparent visualization of decision-making pathways [75].

Decision trees are not typically used for regression (numerical prediction) problems. However, the effectiveness of decision trees in classification has inspired researchers to adapt this method for regression. This adaptation involves assigning ranges to numerical output values and treating them as classes [76].

### Model Assessment

The accuracy of these models was assessed using a goodness-of-fit measure and four different statistical error metrics, which are widely used in water quality modelling studies [29, 30, 32]. These metrics include the coefficient of determination (*R*^2^), root mean squared error (RMSE), mean absolute error (MAE) and mean squared error (MSE), which are calculated as Eqs. 8–11:

8 $${R}^{2}= 1-\frac{\sum_{i=1}^{n}{\left({M}_{i}-{P}_{i}\right)}^{2}}{\sum_{i=1}^{n}{\left({M}_{i}-{M}_{a}\right)}^{2}},$$

9 $$RSME= \sqrt{\frac{1}{n}+\sum_{i=1}^{n}{({M}_{i}-{P}_{i})}^{2},}$$

10 $$MSE= \frac{\sum_{i=1}^{n}{({M}_{i}-{P}_{i})}^{2}}{n} ,$$

11 $$MAE=\frac{1}{n} \sum_{i=1}^{n}\left|{M}_{i}-{P}_{i}\right| ,$$

where ${M}_{i}$ and ${P}_{i}$ represent the measured and predicted water quality parameters, respectively; ${M}_{a}$ denotes the average of the measured values; and $n$ is the total number of paired observed and simulated values.

## Results

### Spatial Clustering Patterns and Homogeneity Analysis

**Fig. 3:**
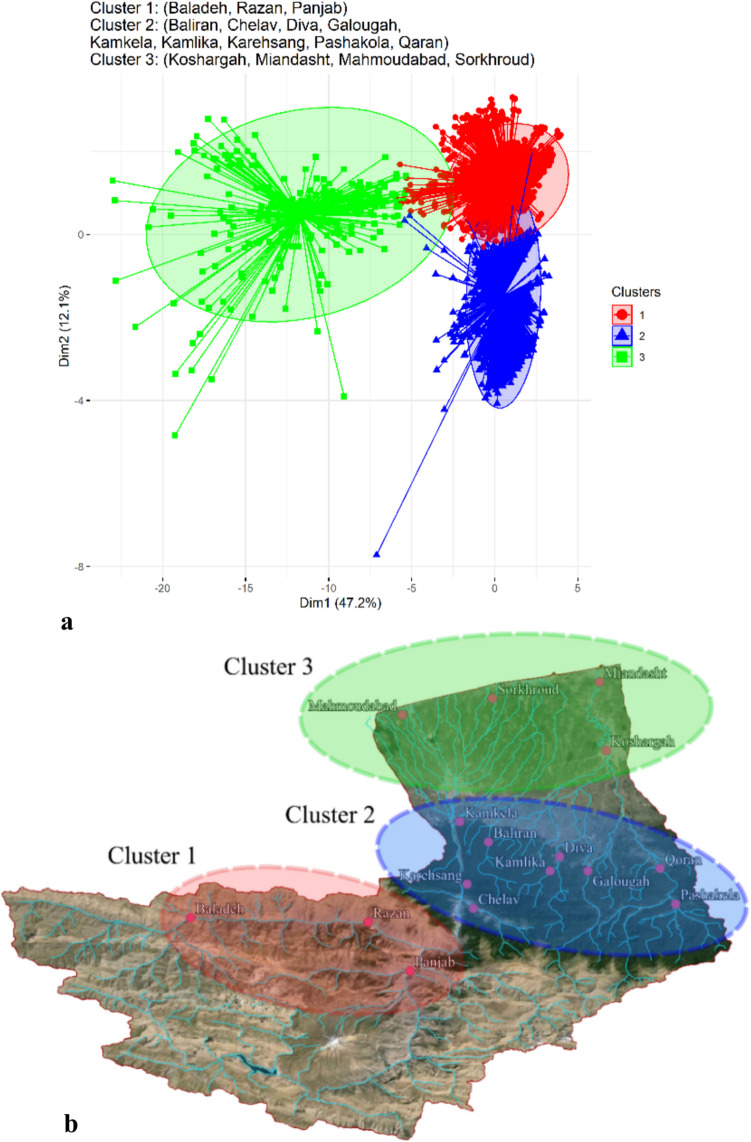
Cluster analysis of water quality stations based on physio-chemical features (**a**) and their spatial location (**b**)

The clustering facilitated modelling of water quality and helped to identify key parameters influencing the water quality index for irrigation purposes. The results reveal three distinct clusters, each exhibiting homogeneous features for both water quality parameters and spatial location (Fig. 3). Cluster 1 represents stations in the natural environments which have a low impact of human activity and are situated in highlands and mountainous regions (Baladeh, Razan, Panjab). Cluster 2 includes stations mostly in forested areas having a low-impact of human activity (Baladeh, Chelav, Diva, Galougah, Kamkela, Kamlika, Karehsang, Pashakola, Qaran) and finally Cluster 3 representing low-land stations with a high anthropogenic impact (Koshargah, Miandasht, Mahmoundabad, Sorkhroud).

### Evaluation of Surface Water Quality

**Fig. 4:**
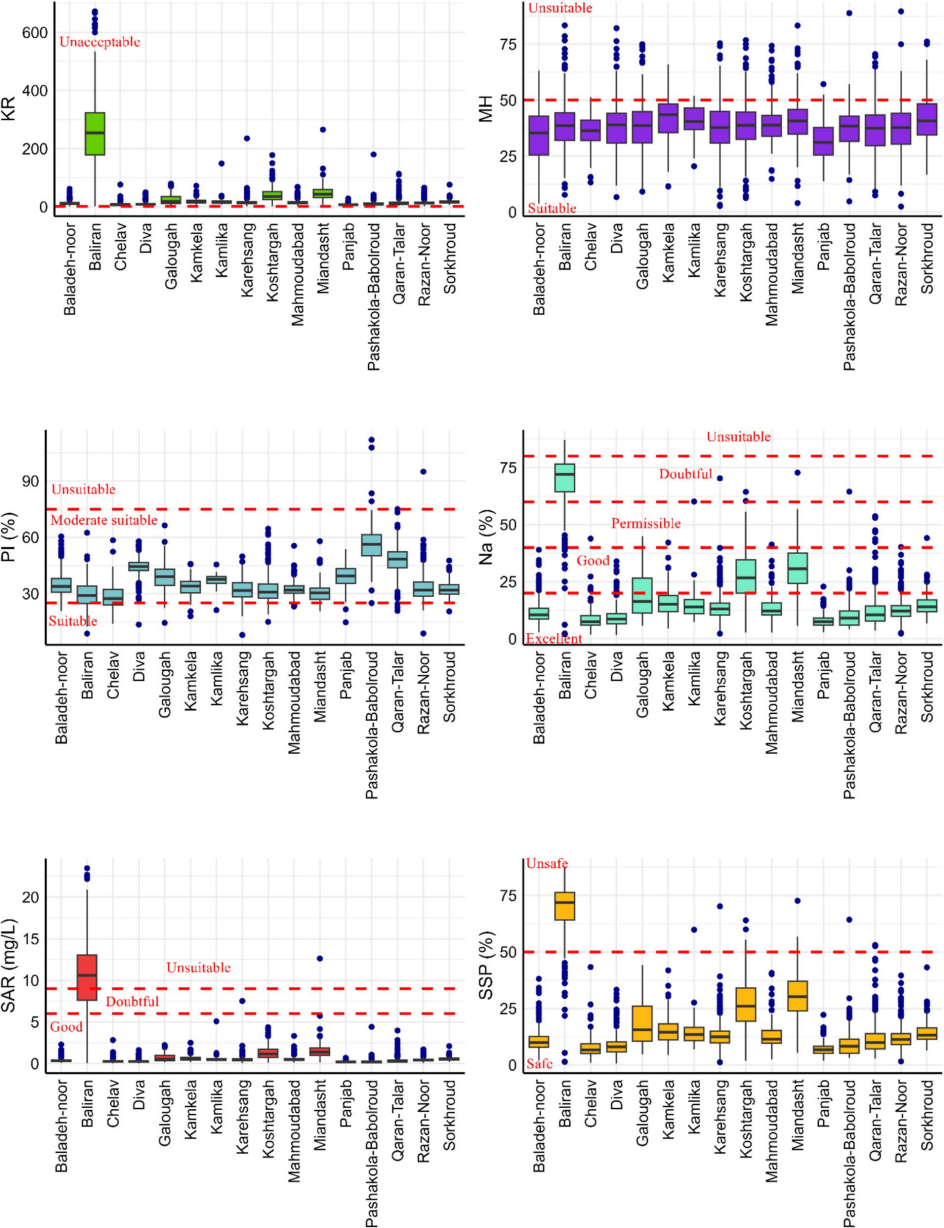
Water quality indices including Kelly’s ratio (KR), Magnesium Hazards (MH), Percent Sodium (Na%), Permeability Index (PI), Sodium Absorption Ration (SAR), and Soluble Sodium Percentage (SPP) variation at hydrometric stations over the studied period

The boxplots in Fig. 4 show how water quality indices vary among the hydrometric stations. The highest variations were identified for MH, SSP and Na%. The MH values ranged from 0.0 to 80.02 mg/L. When assessing the average MH levels of the water at different stations, all stations are in the acceptable class (< 50 mg/lit). Baladeh had the highest variation among all stations (10–65 mg/lit). The PI values were mostly in the range of 20 to 75 at all stations, designating them to class 2 with suitable conditions. The lowest average value (0.12 mg/L) of PI was found at the Chelav station and the highest average value was recorded at the Pashakolah station. The latter had also the largest variation among all stations.

In general, the variability of Na%, SAR and SSP at the Baliran station is different from all other stations. This station had the highest range of these indices. The Na% values lied in the range of 12 and 70% with a minimum average value (12%) at the Panjab station and a maximum (70%) at the Baliran station. Furthermore, the Koshtargah and Miandasht stations had the highest variations among all.

The highest SAR values were registered at the Baliran site with an average value of 10.55. This station is classified in the third SAR category. Unlike Na%, the other stations presented a similar range of SAR values and were in the first SAR class. Like Na% and SAR, the SSP had higher values at the Baliran station compared to the others with an average of 17.8. This station is the only one falling into the class “Unsafe.” All the other stations are classified as “safe” except for single months at the Karehsang, Kamlika, Koshtargah and Miandasht site. In addition, Koshtargah, Miandasht, and Galougah had the highest variation of SSP values while Panjab had the lowest.

### Wilcox Diagram

**Fig. 5:**
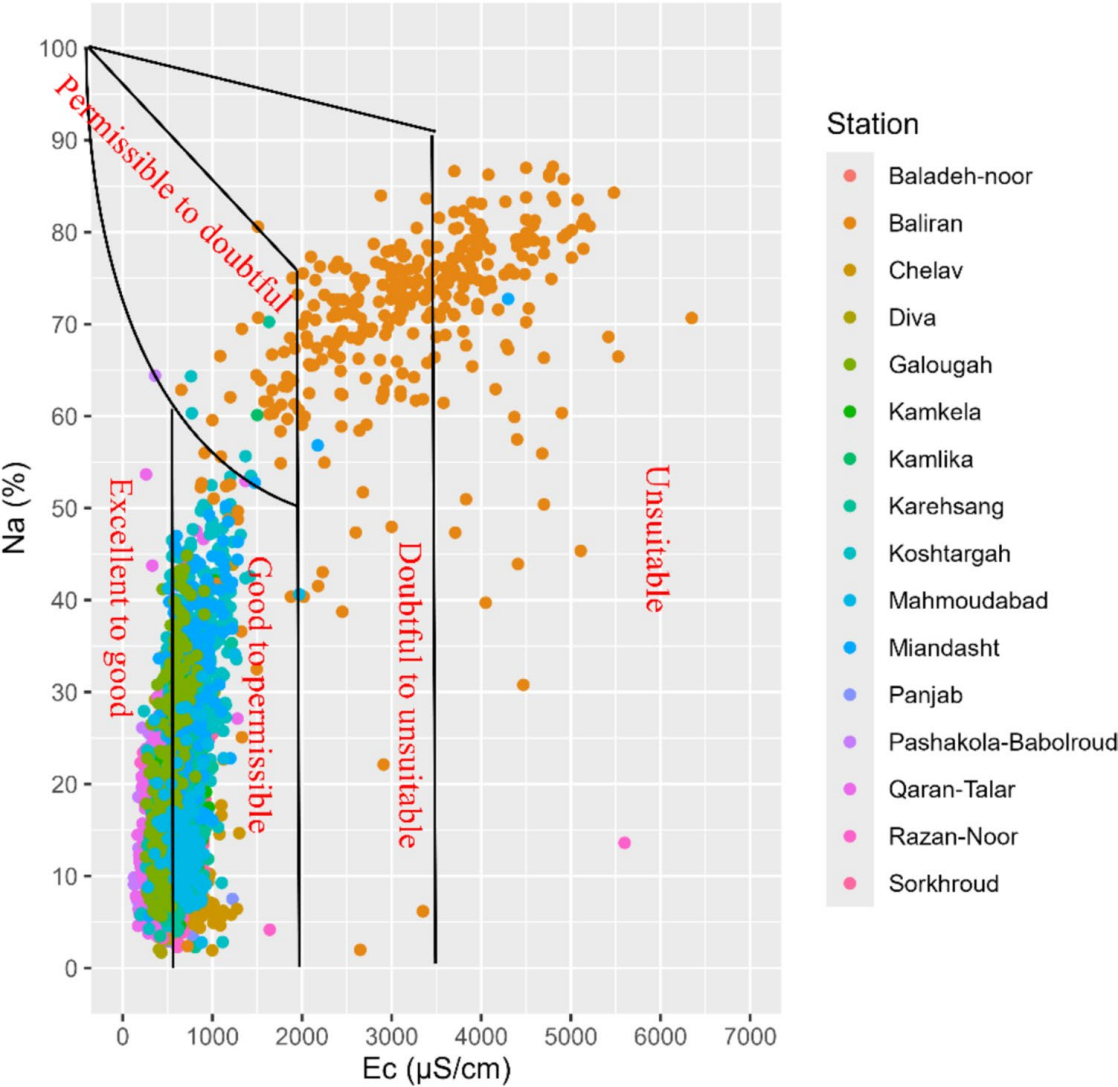
Wilcox diagram for illustrating water quality for irrigation

The Wilcox diagram (Fig. 5) is a tool to evaluate irrigation water quality by considering both Na and EC. When the data is plotted on a graph with EC on the horizontal axis and Na on the vertical axis, the diagram categorises irrigation water quality into five groups: excellent to good, good to permissible, permissible to doubtful, doubtful to unsuitable and unsuitable [23]. The Wilcox Diagram showed that most of the stations (91%) fall into the “good to permissible” category for irrigation, while Baladeh-Noor, Baliran and Chelav were unsuitable. Conversely, the stations of Sorkhroud, Razan-Noor and Qaran-Talar have the best quality in terms of water for irrigation.

### Selecting Critical Water Quality Parameters

The results of the five physio-chemical parameters of water and IWQI at all stations are given in Table 4. All stations were found in the “severe range” (IWQI < 40), restricting the use of water for irrigation to only high-salt-tolerant plants. Irrigation under normal conditions should be avoided.

**Table 4:** The mean and standard deviation of physio-chemical parameters and water quality (Qi and IWQI values)

Stations	EC (μS/cm)	Cl^−^ (meq/L)	Na^+^ (meq/L)	SAR (meq/L)	HCO_3_^−^ (meq/L)	Qi EC	Qi Cl^−^	Qi Na^+^	Qi SAR	Qi HCO_3_^−^	IWQI
Baladeh	549.1 ± 116.6	0.47 ± 0.23	0.60 ± 0.39	0.39 ± 0.23	3.31 ± 0.92	6.95 ± 0.78	6.79 ± 0.00	7.13 ± 0.00	6.57 ± 0.59	7.06 ± 0.00	34.50 ± 0.96
Pashakola	275.5 ± 86.8	0.26 ± 0.17	0.28 ± 0.38	0.25 ± 0.30	2.05 ± 0.72	10.92 ± 2.63	6.79 ± 0.00	7.14 ± 0.00	6.60 ± 0.56	7.06 ± 0.00	38.51 ± 2.66
Panjab	456.2 ± 109.2	0.36 ± 0.21	0.32 ± 0.18	0.22 ± 0.11	2.87 ± 0.75	7.18 ± 0.88	6.79 ± 0.00	7.14 ± 0.00	6.57 ± 0.02	7.06 ± 0.00	34.74 ± 0.88
Chelav	749.0 ± 218.3	0.39 ± 0.18	0.62 ± 0.58	0.34 ± 0.31	3.62 ± 0.86	6.52 ± 0.89	6.79 ± 0.00	7.13 ± 0.01	6.66 ± 1.11	7.06 ± 0.00	34.16 ± 1.34
Diva	427 ± 79.1	0.44 ± 0.29	0.38 ± 0.28	0.27 ± 0.18	3.34 ± 0.68	7.28 ± 0.97	6.79 ± 0.00	7.14 ± 0.00	6.56 ± 0.03	7.06 ± 0.00	34.83 ± 0.98
Razan	613.2 ± 264.4	0.51 ± 0.20	0.73 ± 0.41	0.45 ± 0.24	3.57 ± 1.39	6.81 ± 0.91	6.79 ± 0.00	7.13 ± 0.00	6.53 ± 0.05	7.06 ± 0.00	34.31 ± 0.92
Sorkhroud	659.2 ± 115.7	0.68 ± 0.27	0.96 ± 0.38	0.57 ± 0.22	4.34 ± 1.07	6.63 ± 0.24	6.79 ± 0.00	7.13 ± 0.00	6.56 ± 0.83	7.06 ± 0.00	34.16 ± 0.87
Qaran	367.7 ± 125.2	0.40 ± 0.51	0.47 ± 0.54	0.36 ± 0.33	2.80 ± 0.86	8.50 ± 2.34	6.79 ± 0.00	7.14 ± 0.00	6.64 ± 1.00	7.06 ± 0.00	36.13 ± 2.47
Kamlika	619.1 ± 184.5	0.86 ± 1.66	1.07 ± 1.42	0.66 ± 0.83	4.48 ± 0.82	6.71 ± 0.39	6.79 ± 0.01	7.13 ± 0.01	6.79 ± 1.59	7.06 ± 0.00	34.48 ± 1.26
Karehsang	606 ± 132.4	0.66 ± 0.53	0.82 ± 0.62	0.51 ± 0.38	3.43 ± 0.95	6.75 ± 0.37	6.79 ± 0.00	7.13 ± 0.01	6.55 ± 0.56	7.06 ± 0.00	34.28 ± 0.60
Koshtargah	729.5 ± 226.7	2.12 ± 1.28	2.07 ± 1.30	1.27 ± 0.75	4.17 ± 1.11	6.56 ± 0.88	6.78 ± 0.01	7.12 ± 0.01	8.38 ± 4.44	7.06 ± 0.00	35.90 ± 4.24
Kamkela	625.7 ± 109.5	0.72 ± 0.47	1.00 ± 0.50	0.62 ± 0.31	4.04 ± 0.88	6.70 ± 0.23	6.79 ± 0.00	7.13 ± 0.00	6.59 ± 1.05	7.06 ± 0.00	34.26 ± 1.01
Baliran	3130.1 ± 1076.8	23.07 ± 9.87	21.81 ± 9.54	10.55 ± 4.50	4.61 ± 2.29	1.41 ± 2.27	8.10 ± 4.01	6.99 ± 0.75	9.63 ± 3.46	7.05 ± 0.01	33.18 ± 5.15
Miandasht	793.9 ± 333.9	2.67 ± 2.78	2.61 ± 2.39	1.55 ± 1.08	4.37 ± 1.03	6.37 ± 0.82	6.84 ± 0.89	7.12 ± 0.02	8.83 ± 5.13	7.06 ± 0.00	36.21 ± 4.82
Galougah	511.2 ± 116.3	1.05 ± 0.81	1.01 ± 0.72	0.70 ± 0.48	3.43 ± 0.65	7.04 ± 0.82	6.78 ± 0.00	7.13 ± 0.01	6.70 ± 1.63	7.06 ± 0.00	34.72 ± 1.75
Mahmoudabad	704.6 ± 178.9	0.76 ± 0.79	1.01 ± 0.82	0.56 ± 0.38	5.14 ± 1.20	6.57 ± 0.63	6.79 ± 0.00	7.13 ± 0.01	6.68 ± 1.35	7.05 ± 0.00	34.22 ± 1.26

The mean EC ranged from 275.5 to 3130.1 μS/cm, indicating a high salinity at Baliran, Miandasht and Chelav, respectively. The mean chloride concentrations also varied between 0.26 and 23.07 meq/L among the stations (Table 1). The concentrations of Na^+^ were relatively low with averages ranging from 0.28 to 21.81 mg/L (Table 1). The highest mean Na^+^ concentration (21.81 mg/L) was measured at the Baliran station. The average concentration of HCO_3_^−^ ranged from 2.05 to 5.14 mg/L.

**Fig. 6:**
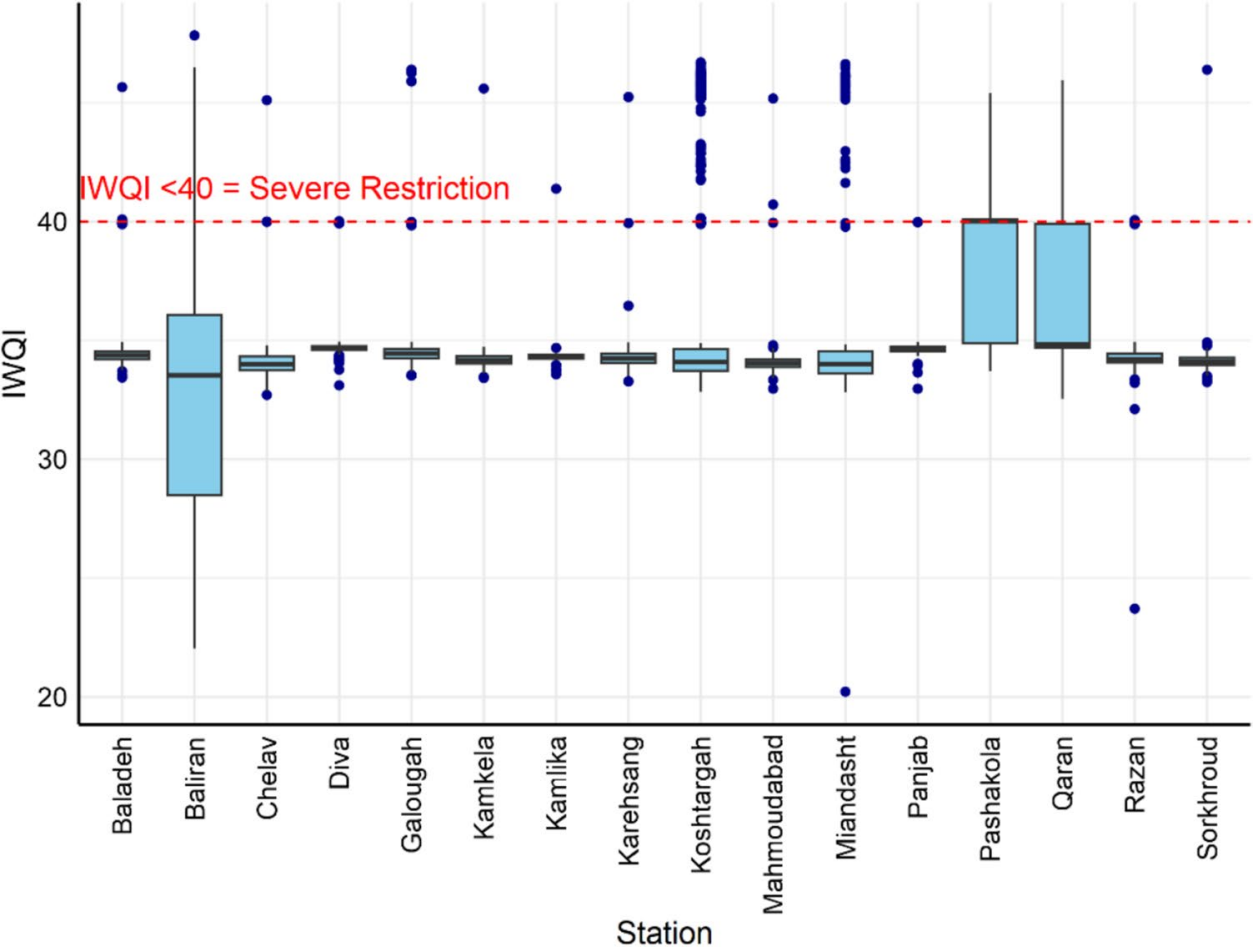
Variability of the IWQI for water quality among all observation stations

All sites exhibit a severe restriction according to the IWQI with values of less than 40. The Baliran, Qaran, and Pashakola stations had the highest variability of IWQI (Fig. 6). These differences suggest that local contamination sources play a more significant role than regional factors for water quality.

The Baliran station had the lowest median IWQI value while Pashakola and Qaran had the highest one and the rest of the stations ranged between 34 and 36.

### Significant Parameters for IWQI Modelling

Using the stepwise regression method, the contribution of different parameters to the IWQI was explored for each cluster. As a result, the most important water quality parameters for the IWQI remained for modelling (Table 5) and the rest of them were excluded from the dataset. The Pr (>|t|) values for the independent parameters are presented in Table 3. If Pr (>|t|) is below the threshold, the null hypothesis is accepted, indicating a statistically significant difference. Conversely, if Pr (>|t|) exceeds the threshold, the null hypothesis is rejected, suggesting that the difference is not statistically significant.

**Table 5:** Parameter’s coefficient of using stepwise regression for different Clusters 1, 2, and 3

**Cluster1**	Estimate	Std. Error	*t*-value	Pr(>|t|)
Intercept	33.88	0.59	57.23	< 2.00E-16***
EC	−0.21	0.01	−15.28	< 2.00E-16***
SAR	−3.14	0.77	−4.08	< 4.96E-05***
TDS	−0.01	0.00	−4.55	6.07E-06***
Q	0.03	0.00	5.31	1.38E-07***
pH	0.34	0.07	4.98	7.42E-07***
Ca	−2.40	0.50	−4.77	2.09E-06***
Mg	−2.52	0.50	−5.00	6.65E-07***
SOC	2.56	0.48	5.30	1.41E-07***
Tem_H	0.00	0.00	1.74	8.15E-02
**Cluster2**				
Intercept	33.86	0.91	37.33	< 2.0E-16***
Cl	16.59	1.04	15.91	< 2.0E-16***
EC	−0.62	0.05	−12.48	< 2.0E-16***
SAR	−0.64	0.09	−7.11	1.4E-12***
Q	−0.01	0.00	−7.72	1.6E-14***
pH	0.61	0.11	5.53	3.5E-08***
Ca	−0.32	0.08	−3.99	6.6E-05***
Mg	−0.31	0.08	−3.76	1.8E-04***
SOC	0.42	0.07	6.15	8.8E-10***
Tem_H	−0.01	0.00	−8.65	< 2.0E-16***
Tot_H	0.00	0.00	3.06	0.002207**
**Cluster3**				
Intercept	29.83	0.63	47.20	< 2.00E-16***
EC	−1.41	0.45	−3.15	1.66E-03**
HCO_3_^−^	−21.76	11.62	−1.87	6.16E-02
SAR	9.55	0.64	14.91	< 2.00E-16***
TDS	0.01	0.01	1.56	1.18E-01
Q	0.01	0.00	2.04	4.15E-02*
SO4	0.26	0.18	1.45	1.49E-01
Ca	4.23	0.48	8.88	< 2.00E-16***
Mg	4.19	0.49	8.59	< 2.00E-16***
SOC	−3.11	0.46	−6.73	2.85E-11***
Tot_H	0.01	0.00	2.69	7.25E-03**

Note: Significance symbols: 0'***', 0.001'**', 0.01'*',0.05'.', 0.1 ‘ ‘

Considering a threshold value of 0.05 and the null hypothesis, the only unimportant input parameter was total hardness in Cluster 1. Therefore, the IWIQ variation in this cluster is explained by eight predictor parameters namely: EC, SAR, TDS, Q, pH, Ca, Mg, and SOC.

In Cluster 2, there are no unimportant input parameters among the studied ones, and all parameters are included for further analysis. In Cluster 3, the insignificant parameters are HCO_3_^−^, TDS and SO_4_^2−^, with a corresponding Pr(>|t|) larger than 0.05.

A correlation analysis was performed to determine the relationship among the most effective water quality parameters and the IWQI within the Clusters 1, 2, and 3 (Table 3).

**Fig. 7:**
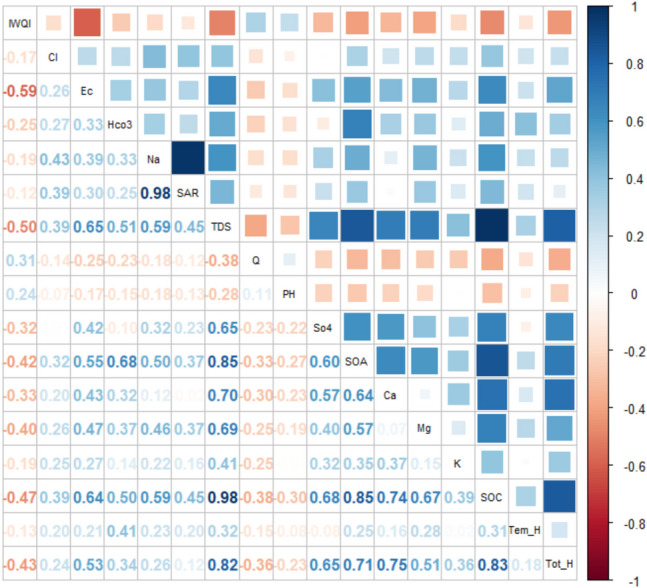
Pearson correlation coefficient of water quality parameters with IWQI in cluster 1

In cluster 1 (Fig. 7), the results indicated a strong positive correlation between Na and SAR, as well as between TDS, some cations and total hardness. There are also positive strong correlations between TDS, the sum of anions and cations indicating that TDS can be estimated using these two parameters. In addition, strongly and positively correlating parameters were the TDS, Tot_H and also TDS and sum of anions.

**Fig. 8:**
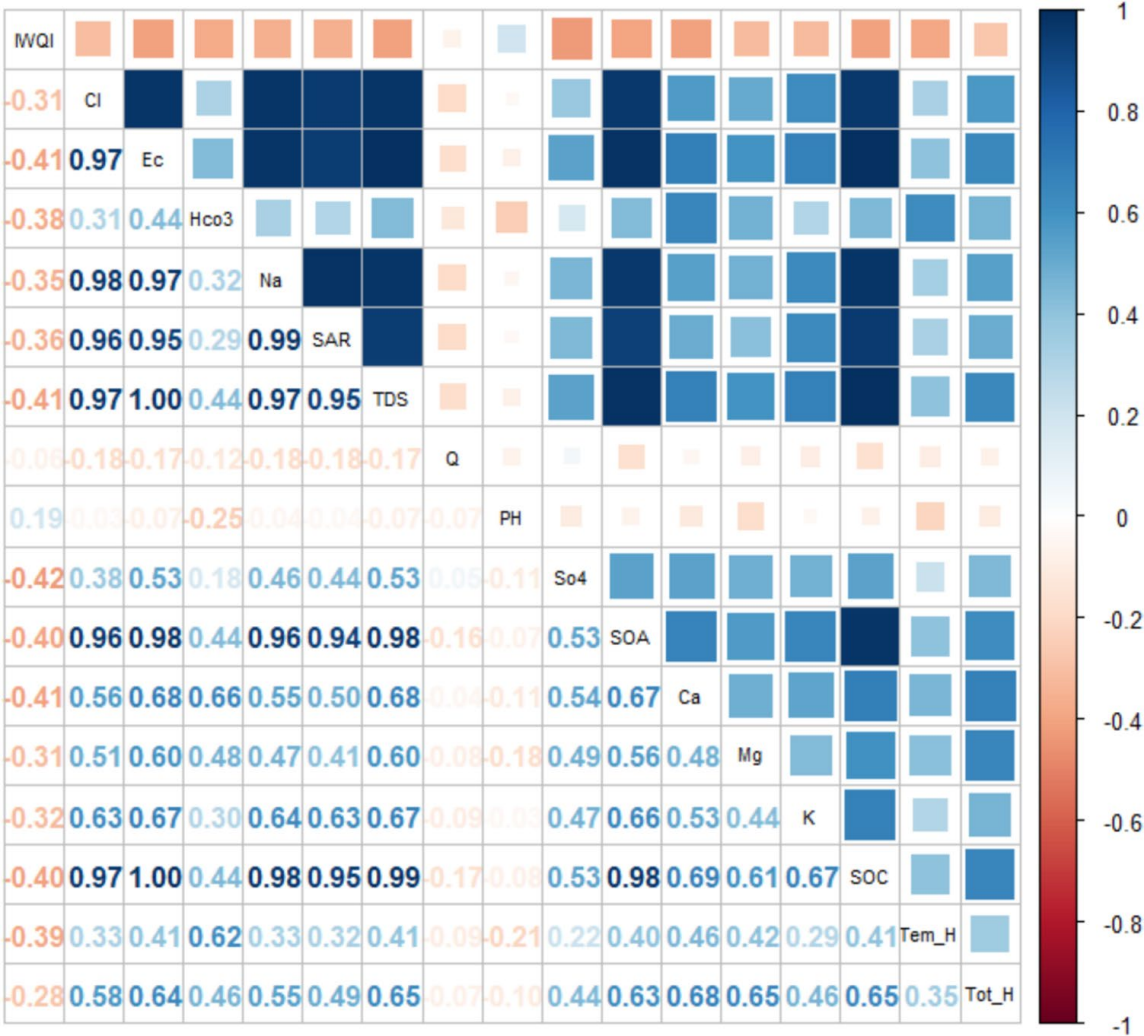
Pearson correlation coefficient of water quality parameters with IWQI in Cluster 2

In cluster 2 (Fig. 8), high positive correlations were found between Cl and some other parameters such as EC, Na, SAR, TDS, SOA, and SOC.EC is positively correlated with TDS (1.0), Na (0.97), Cl^−^(0.97), SAR (0.95), Ca (0.68), Mg (0.60), SO_4_ (0.53), and HCO_3_^−^ (0.44). The large variability of EC is influenced by lithology, land use and human activity [77]. In cluster 1, Na is positively correlated with Na (1.0), SAR (0.99), TDS, and Cl. A high correlation between these parameters has also been reported by other studies [77]. EC and TDS usually showed positive correlations with Cl, SO4 and Na. In addition, IWQI has a moderately negative correlation with SO4, EC, TDS, Ca, SOA, and SOC.

**Fig. 9:**
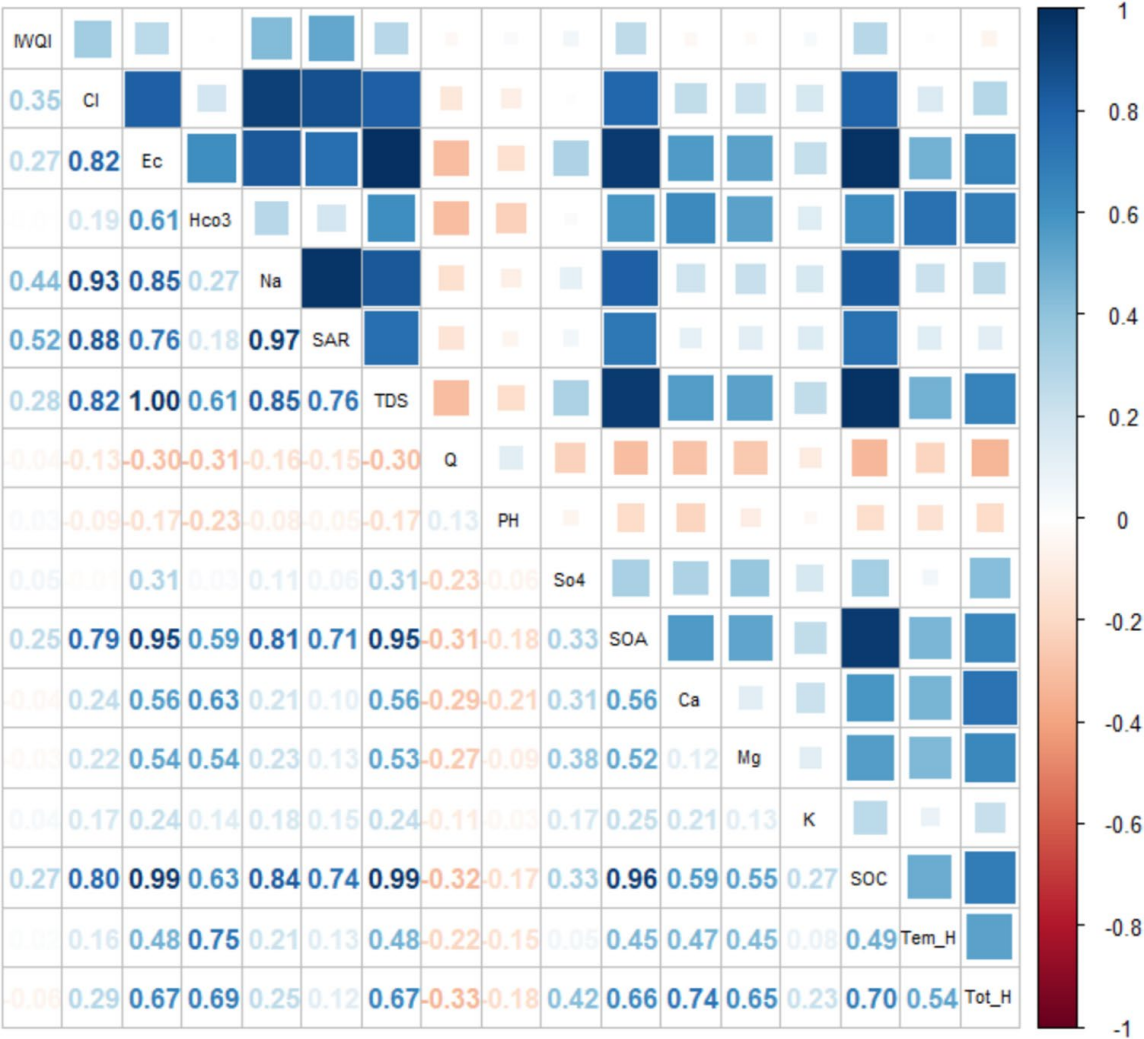
Pearson correlation coefficient of water quality parameters with IWQI in Cluster 3

In cluster 3 (Fig. 9), positive correlations between the Cl and some parameters such as EC (0.82), Na-P (0.93), SAR (0.88), TDS (0.82), Sum-A (0.79), Na (0.93), and Sum-K (0.80) were determined. In addition, moderately positive correlations (*r* = 0.52 and *r* = 44; *P* > 0.05) were found between the IWQI and Na-P, Na and SAR. Furthermore, positive correlations between the IWQI and Cl, EC, TDS, Sum-A, Sum-C (*r* = 0.35, *r* = 0.27, *r* = 0.28, *r* = 0.25, *r* = 0.27; *P* > 0.05) and negative correlation between EC and Q, Q and hardness-To, Q and Sum-K (*r* = −0.30, *r* = −0.33,* r* = −0.32) were determined but the results were not statistically significant.

### Principal Component Analysis

In each cluster, the relationship among monthly water quality parameters and IWQI were further assessed through a Principal Component Analysis (PCA) to identify underlying patterns and correlations within the data.

**Fig. 10:**
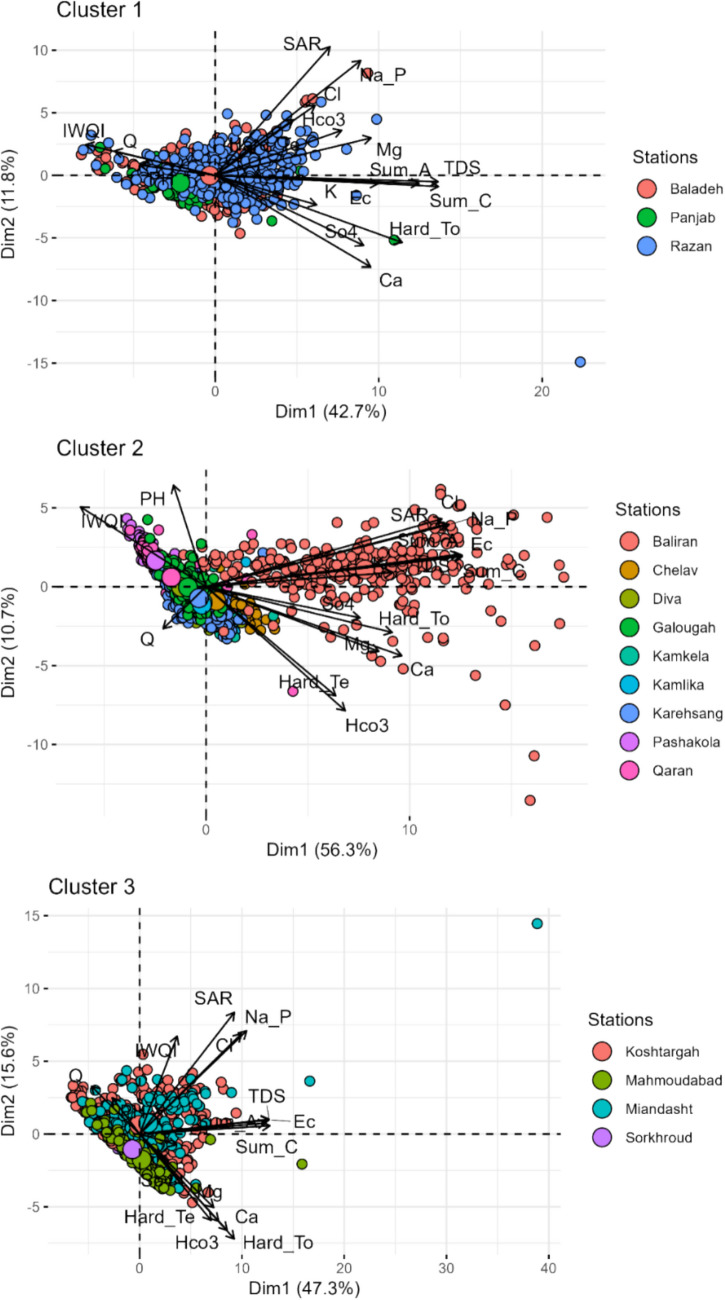
Principal component analysis of water quality parameters and related stations in different months

In cluster 1, which includes monthly water quality data from three stations (Baladeh, Razan and Panjab), the PCA revealed a significant relationship between the IWQI and other water quality parameters (Fig. 10).

IWQI exhibits a negative relationship with Dim.1 (−0.564), suggesting that higher values of IWQI correspond to lower scores. This indicated that elevated IWQI values were associated with poorer water quality having higher TDS, Na and SO_4_. Conversely, IWQI showed a positive relation with Dim.2 (0.175), implying that higher IWQI values are linked to higher Ca, HCO_3_^−^ and temporary hardness.

Within cluster 2, IWQI demonstrated a negative correlation with the Dim.1 suggesting that lower IWQI values corresponded to higher scores on Dim.1. This relationship implied elevated levels of TDS, Na, SO4 and some of anions and cations in hydrometric stations associated with lower IWQI values. Conversely, IWQI exhibited a positive correlation with Dim.2, indicating that higher IWQI values are linked to increased Ca, HCO_3_^−^ and temporary hardness.

The PCA analysis of cluster 3 revealed distinct positive and negative correlations of IWQI with various water quality parameters. IWQI demonstrated a strong positive correlation with Cl (0.776), Na (0.815), and TDS (0.986) meaning that high IWQI values indicate elevated concentrations of these parameters. Conversely, the IWQI exhibited a moderate and negative correlation with EC (−0.986). Additionally, the IWQI showed a moderate and positive correlation with HCO_3_^−^ (0.516).

### Model Evaluation

The proposed framework first clusters monitoring stations based on physiochemical characteristics, creating spatially homogenous groups. For each cluster, predictive ML models were trained independently to capture local variations of water quality. This spatially adaptive approach improved the accuracy compared to using a single and global model for the entire watershed.

The performances of the models for training and testing stages are presented in Table 6. The XGBoost showed the best and SVM the worst performance for modelling water quality (IWQI) in the mentioned clusters, respectively.

**Table 6:** Model evaluation statistics of ML models for IWQI in different clusters

	Training stage				Testing stage			
**Cluster 1**	*R*2	RMSE	MAE	MSE	*R*2	RMSE	MAE	MSE
RF	0.92	0.30	0.03	0.09	0.87	0.20	0.05	0.04
BRT	0.81	0.45	0.07	0.20	0.64	0.41	0.11	0.17
ET	0.96	0.22	0.02	0.05	0.89	0.18	0.04	0.03
XGBoost	1.00	0.02	0.01	0.00	0.98	0.07	0.02	0.00
SVM	0.61	0.74	0.15	0.55	0.70	0.27	0.08	0.07
DT	0.78	0.48	0.17	0.23	0.70	0.28	0.16	0.08
**Cluster 2**	Training stage				Testing stage			
RF	0.98	0.35	0.08	0.12	0.93	0.68	0.14	0.46
BRT	0.98	0.40	0.12	0.16	0.94	0.59	0.15	0.35
ET	0.99	0.22	0.03	0.05	0.96	0.49	0.08	0.24
XGBoost	1.00	0.05	0.02	0.00	0.95	0.54	0.09	0.29
SVM	0.72	1.43	0.61	2.06	0.63	1.56	0.67	2.43
DT	0.92	0.74	0.29	0.55	0.89	0.84	0.31	0.70
**Cluster 3**	Training stage				Testing stage			
RF	0.99	0.34	0.08	0.12	0.99	0.37	0.14	0.14
BRT	0.97	0.64	0.15	0.41	0.99	0.39	0.15	0.15
ET	0.99	0.27	0.03	0.07	1.00	0.06	0.03	0.00
XGBoost	1.00	0.02	0.02	0.00	1.00	0.10	0.06	0.01
SVM	0.63	2.36	1.05	5.55	0.67	2.54	1.16	6.44
DT	0.96	0.76	0.35	0.58	0.98	0.61	0.35	0.37

In cluster 1, the models differed in terms of NSE, RMSE, MAE, and *R*^2^. The SVM model exhibited the weakest overall performance among all models in the training stage (Table 6, Fig. 6). Comparatively, the XGBoost model outperformed all models during both the validation and testing phases. In the training stage, the XGBoost model achieved *R*2, RMSE, MAE, and MSE values of 1.0, 0.02, 0.01, and 0.00, respectively.

In cluster 2, XGBoost demonstrated the highest prediction accuracy with an *R*^2^ score of 1, closely followed by ET, BRT, and RF, which exhibited high accuracies and better model fitting on the training and testing data, as evidenced by their low MAE, MSE, RMSE, and RMSPE values, and high *R*^2^ scores. Although these models showed strong performance, SVM exhibited a lower performance on the training and test data with a lower *R*^2^ scores and higher MAE, MSE, RMSE, and RMSPE values compared to other models.

In cluster 3, the different models had a similar performance with small variations in NSE, RMSE, MAE, and *R*^2^. However, the XGBoost model had the best performance across four metrics of the training stage. The XGBoost and ET models exhibited an equal accuracy with an R^2^ score of 1 in the testing stage. Additionally, the RF, BRT and DT models showed a high and similar level of performance to XGBoost, achieving *R*^2^ scores of 0.99, 0.99, and 0.98, respectively. As in previous clusters, the SVM exhibited an acceptable performance with moderate metric values. It is important to recognise that a model’s performance on training data may not be generalised. Thus, additional evaluation of the models’ overall performance using validation and test data is essential to determine the most appropriate model for predictive tasks [64]. The comparisons of these six ML models are further shown in Fig. 9 for the results of water quality prediction and performance.

**Fig. 11:**
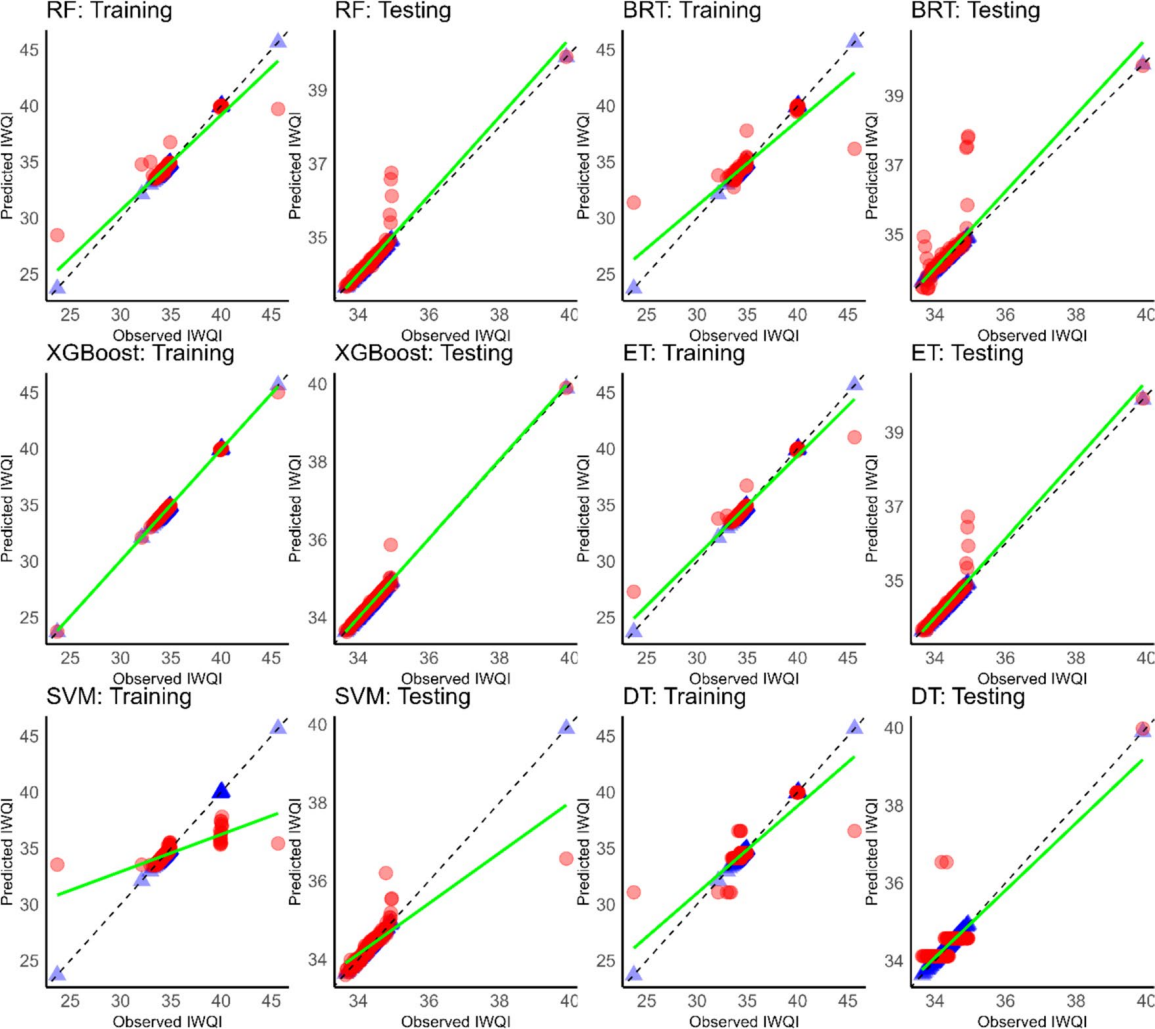
Scatter plot of predicted (red dots) versus measured IWQI (blue dots) using ML models of Cluster 1. The green line is the best fit through predicted values and the dashed line is the perfect prediction where simulated values = observed values

**Fig. 12:**
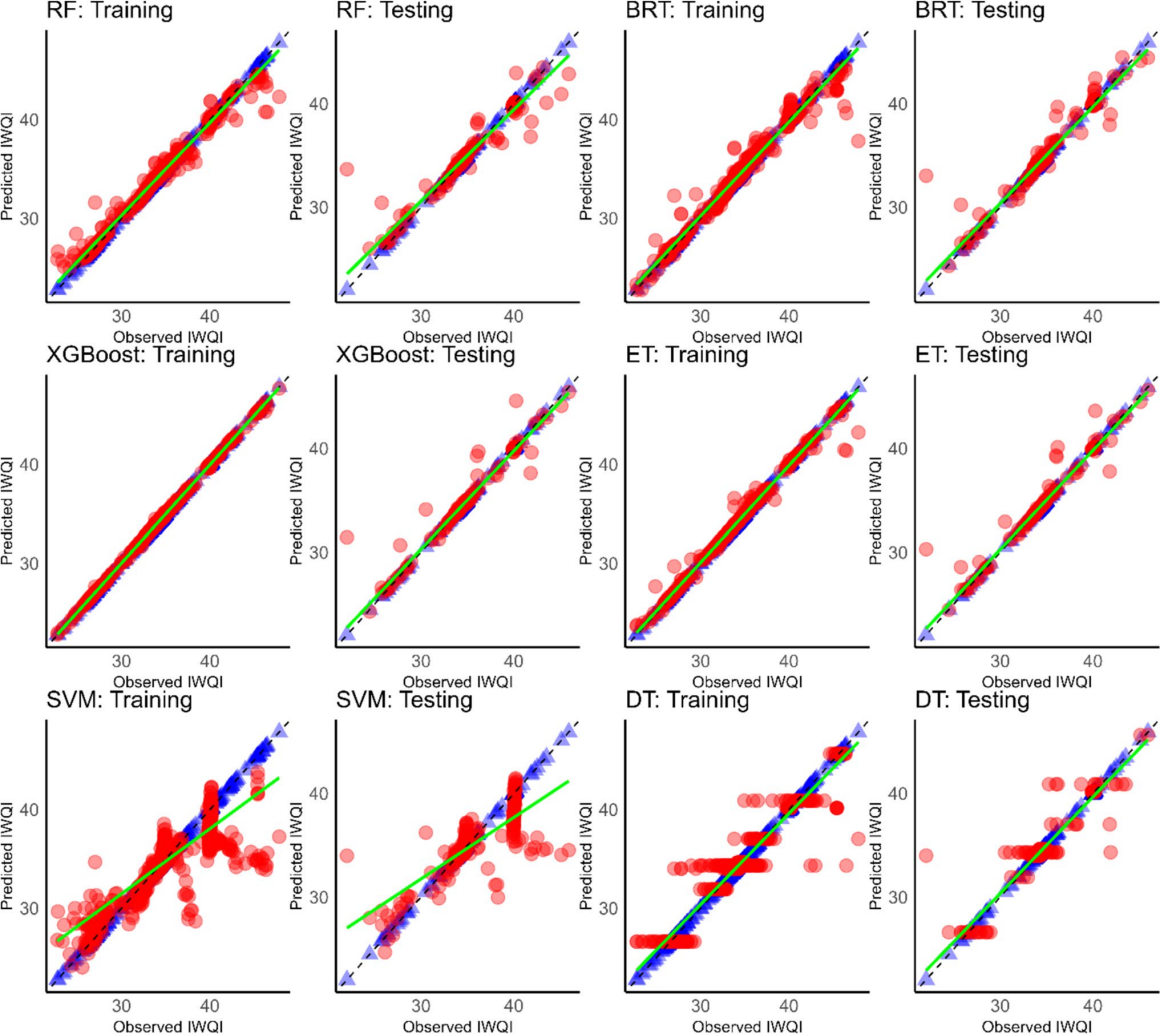
Scatter plot of predicted (red dots) versus measured IWQI (blue dots) using ML models of Cluster 1. The green line is the best fit line through the predicted values and the dashed line is the perfect prediction where simulated values = observed values

**Fig. 13:**
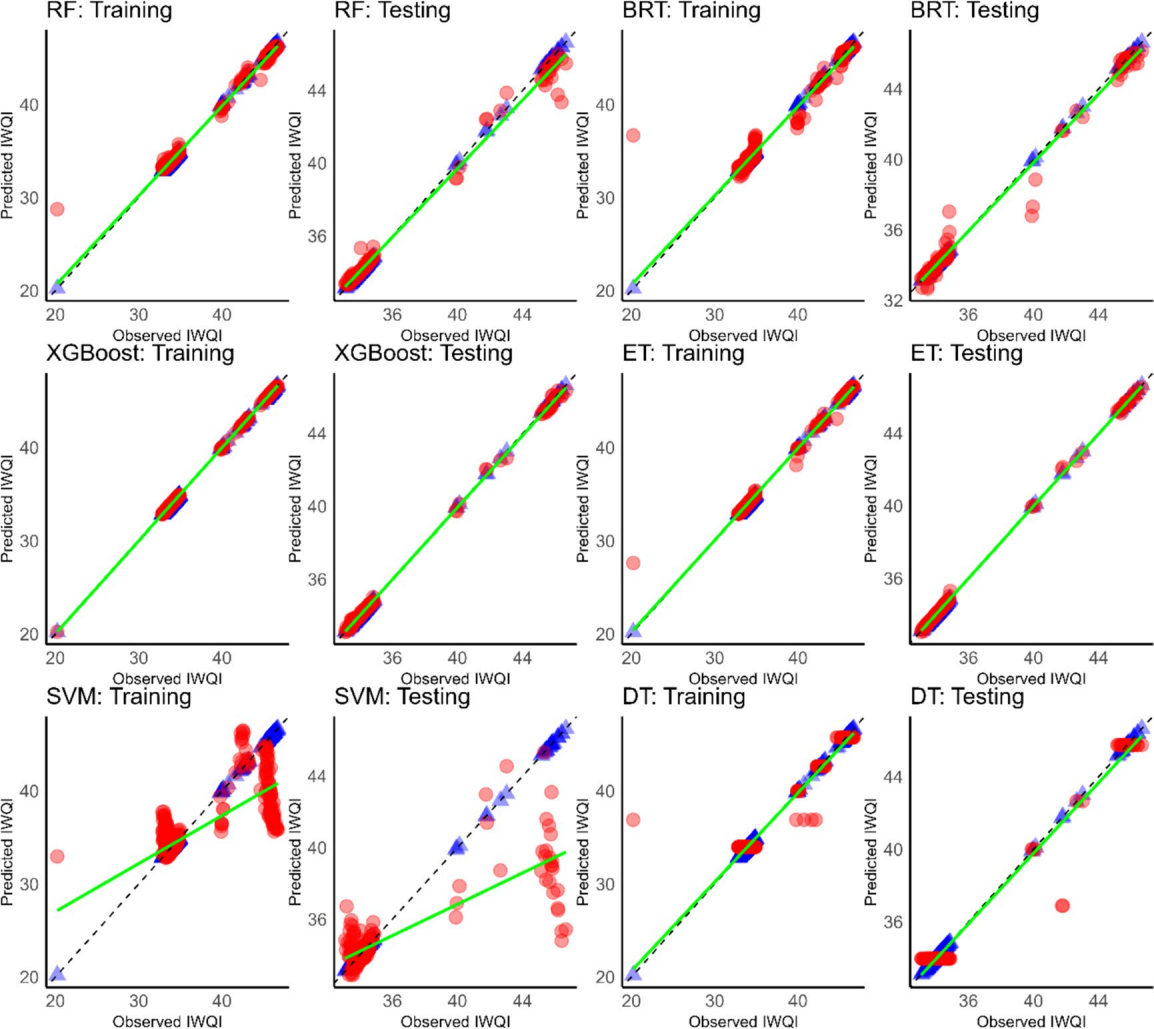
Scatter plot of predicted (red dots) versus measured IWQI (blue dots) using ML models of Cluster 1. Th green line is the best fit line through the predicted values and the dashed line is the perfect prediction where simulated values = observed values

The visualisation of the relationship between measured and predicted values is a crucial aspect in assessing model performance and accuracy. Figures 11, 12, and 13 provide a visual representation of the predictive abilities of the applied six ML models. The blue and red points are depicted as measured and predicted data, respectively.

As shown in Fig. 11, XGBoost showed a better prediction power than the other models in cluster 1. Similar findings were obtained for cluster 2 (Fig. 12). XGBoost also outperformed the other models in terms of predictive accuracy, showing higher *R*^2^ values and the lowest RMSE and MAE values.

In addition, in cluster 3 (Fig. 13), XGBoost continued to outperform. Additionally, a decrease in performance and accuracy was noted with SVM in cluster 3 compared to the previous ones.

**Fig. 14:**
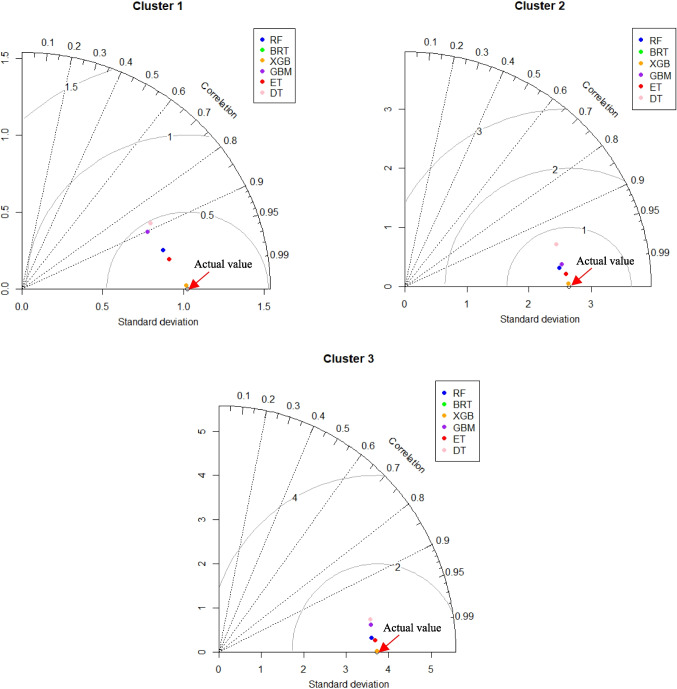
Taylor diagrams for the prediction of the IWQI of the different Clusters 1, 2, and 3

To conduct additional testing and verifying of the prediction accuracy of six ML models, a Taylor diagram (Fig. 14) was employed to statistically assess the agreement between the values of observed and predicted IWQI. The correlation coefficient (CC), standard deviation (SD) and centred root-mean-square error (CRMSE) are combined in a polar coordinate diagram using the triangular cosine relationship among them. The analysis of prediction was performed based on the distribution of three evaluation metrics in this diagram. Within cluster 1, 2, and 3, XGBoost exhibited a superior prediction performance. These findings agreed with earlier results, confirming that the XGBoost model produced more precise prediction outcomes compared to other models.

### The Importance of Local Parameters

**Fig. 15:**
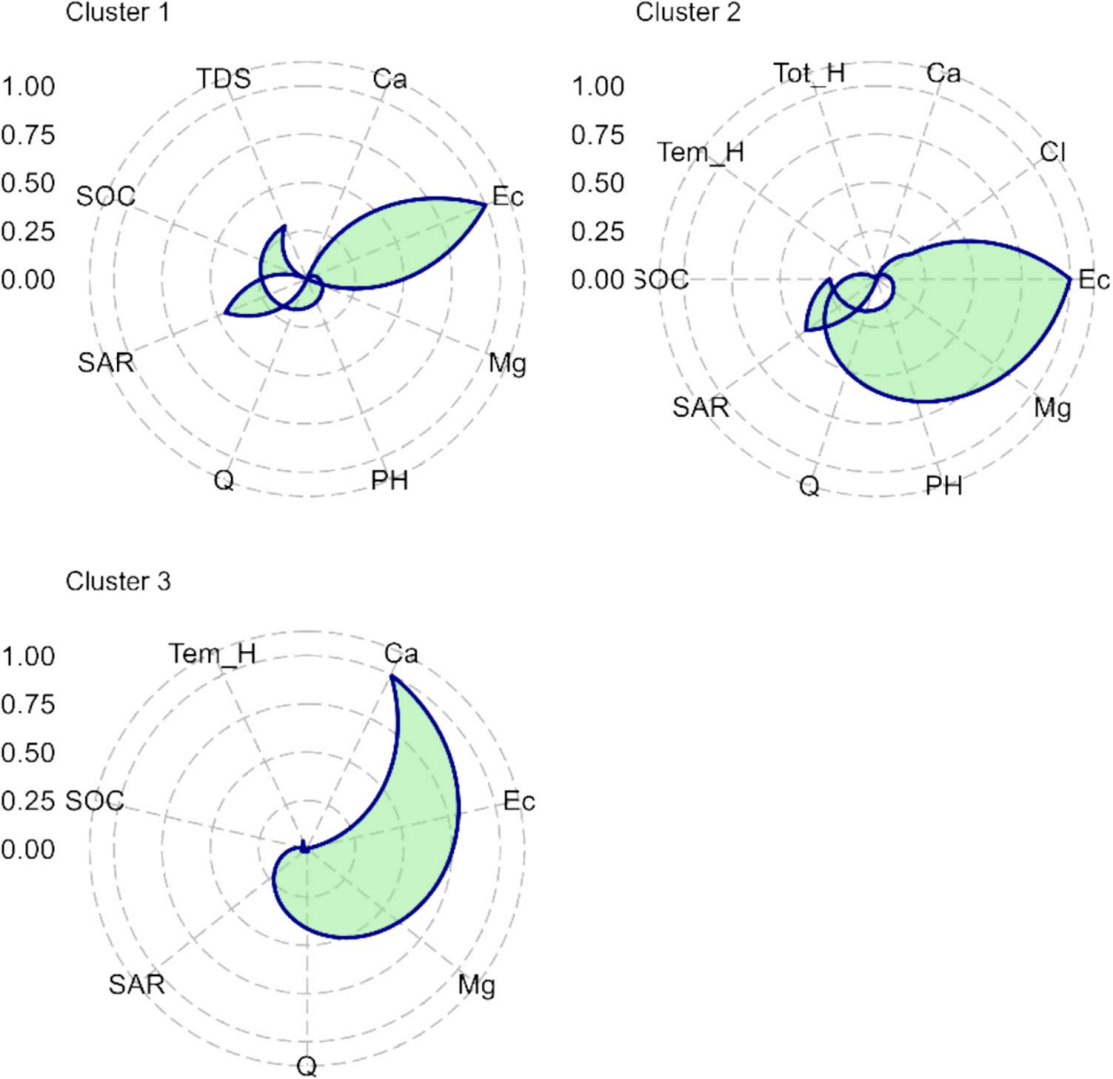
Radar plot of the importance of parameters for modelling using XGBoost

For the most accurate model (XGBoost), the impact of essential water quality parameters on the predictive capability was calculated and, thus, their contribution to the predictive model derived. The results of the relative importance in each cluster are illustrated in Fig. 15. The importance scores in cluster 1 showed that EC, SAR, TDS, SOC, Mg, Ca, Q, and pH are the most relevant parameters. In cluster 2, the parameters were ranked in the following decreasing order of importance: EC, SAR, SOC, Cl, Tot_H, Q, Mg, Ca, PH, and Tem_H. In Cluster 3, the decreasing rank of importance was Ca, Tem_H, SOC, Ec, Q, Mg, and SAR.

## Discussion

Accurate water quality predictions play a crucial role in providing a reliable basis for managing river water quality and pollution management. The performance and effectiveness of water quality prediction models are influenced by both the input data and the model characteristics [78]. However, spatial variations in water quality data are a challenge to develop an overall model for water quality prediction.

### River Water Suitability for Irrigation

To assess the water quality of the Haraz and Babolroud rivers for irrigation purposes, several indices such as IWQI, KR, Na (%), MH, SAR, SSP, and PI were applied monthly. These indices were used to determine the suitability of physiochemical features for irrigation in the Amol-Babol plain, the main centre of agriculture in this region (especially rice cultivation with traditional submergence farming system). This region has experienced an agriculture that relies on irrigation already for a long time, owing to its conducive environmental factors such as fertile soils, topography, good access to both surface and groundwater reservoirs and a notably high level of precipitation. Consequently, river water quality is a vital issue in this region as it has a direct impact on agricultural production, public health and food security. According to the IWQI, all monitoring stations were in the category “severe restrictions” (IWQI < 40), with certain months falling into the category of “high restrictions” (40 < IWQI < 50). Based on the KR, all stations in all months were classified as unacceptable, exhibiting excess sodium in water that strongly limits its application in agriculture. Conversely, the MH index consistently calculated values below 50, suggesting the water's suitability for irrigation and indicating an absence of a magnesium hazard. For the PI index, all stations were grouped in the class “moderately suitable.” The results even indicated that all stations, except Kostrgah and Miandasht, were excellent or good based on the Na levels. Kostrgah and Miandasht fell into the category “good,” while Baliran was classified as doubtful. Considering the SAR, all stations showed a “good” water quality for irrigation, while Baliran was classified as unsuitable for irrigation. The result of the last IWQI, the SSP, indicated that only the Baliran station was in the class “unsafe,” while all other stations indicated “safe” conditions. In general, the stations situated in plain area such as Koshtargah and Miandasht exhibited a reduced irrigation water quality. These stations are positioned at the outlet points of the Haraz and Babolroud watersheds. These are rivers that cross major urban centres and densely populated regions, thereby conveying elevated concentrations of pollutants (suspended and dissolved materials) stemming from anthropogenic sources including agricultural and industrial practices.

### Model Performance in Different Clusters

Table 6 illustrates the variation in model performance across different clusters, which can be attributed to differences in observed data patterns, interactions among WQ parameters, and distribution characteristics unique to each cluster and model features [32]. While all models demonstrated acceptable performance except for SVM, XGBoost exhibited superior performance particularly in clusters 1 and 3 when compared to other models such as RF, ET, and ANN. As a gradient-boosted decision tree algorithm, XGBoost effectively captures nonlinear relationships and complex feature interactions through its iterative learning framework [64]. In Clusters 1 and 3, where data patterns appear more homogeneous and structured, XGBoost demonstrates exceptional performance, achieving higher *R*^2^ values and minimal RMSE and MSE. Conversely, in Cluster 2, although XGBoost still outperforms other models, its testing performance declines slightly, most likely due to increased data heterogeneity, noise, or more complex distributions. In addition, XGBoost has been effectively applied in other river water quality studies. For example, Lu et al. [42] introduced a novel hybrid model combining XGBoost with the CEEMDAN denoising technique for improved short-term water quality prediction. They reported that the CEEMDAN-XGBoost model excelled in predicting pH, turbidity and fluorescent dissolved organic matter using hourly data. Furthermore, Xu et al. [79] utilized XGBoost to analyse factors affecting the spatio-temporal variation of water quality, specifically focusing on the potassium permanganate index, total phosphorus, and total nitrogen in a fragile karst watershed. Our study demonstrates that XGBoost is effective in identifying potential water quality hot spots in unmonitored areas, offering valuable insight for improving water quality management. This model enhances model performance by refining weak learners’ residuals, which are the differences between predicted and actual values, through an iterative process [42]. Its effectiveness with sparse data is due to a sparsity-aware split-finding approach [80]. Additionally, XGBoost stands out for its unique objective function and the flexibility it offers in choosing loss functions. Its ability to process large datasets swiftly and efficiently is attributed to block technology and the use of CPU multithreading for parallel processing. These features, combined with continuous algorithmic improvements, make XGBoost highly effective for water quality studies, where precision and efficiency are critical.

However, while XGBoost often demonstrates superior performance, other models have occasionally outperformed it in specific contexts. For instance, Raheja et al. [81] compared the prediction capabilities of different models including DNN, Gradient boosting machine (GBM) and XGBoost. They found that DNN can provide a better accuracy and robustness for water quality prediction than XGBoost. Similarly, other researchers found that SVM and Multilayer Perceptron (MLP) provided a higher prediction accuracy than XGBoost in predicting multiple water quality parameters [82, 83].

### Topography and Land Use Effects

The studied watersheds can be categorised into three distinct clusters based on spatial characteristics, particularly topography and LULC. Cluster 1 is primarily mountainous with steep slopes and predominantly natural land covers such as poor vegetation covers, bare soil and outcrop, characterised by relatively low anthropogenic disturbance. Cluster 2 represents a transitional landscape with highly complex and heterogeneous land use, including forest areas, agricultural lands, residential zones, mining activities, and dam constructions, all situated on moderately sloped terrain. Cluster 3, by contrast, is a lowland plain dominated by intensive rice cultivation and residential settlements. Figure 10 illustrates the spatial relationships between the spatial location of the WQ stations and water quality parameters across the clusters. It is evident that certain WQ parameters are associated with specific stations within each cluster. These spatial dependencies significantly influence the water quality parameters, which may affect the performance of the models. In Clusters 1 and 3, where land use and topography are more homogeneous, ML models achieved better predictive accuracy, evidenced by higher *R*^2^ values and lower RMSE, MSE, and MAE values (Table 6). The relative uniformity of land cover in these clusters likely contributes to more consistent pollutant loading patterns, simplifying the modelling of observed data. Conversely, Cluster 2 exhibited the lowest model performance, likely due to the high degree of spatial heterogeneity and anthropogenic disturbance. The presence of multiple pollution sources, including agricultural and urban runoff, results in complex and nonlinear water quality dynamics that are challenging for data-driven models to accurately capture. This is especially evident at the Baliran, Qaran and Pashakola stations, which showed higher IWQI values, highlighting the potential link between complex land use and model accuracy. Previous studies have demonstrated that LULC significantly influences the performance of ML models in water quality prediction [84, 85]. In this study, land use was indirectly incorporated into the modelling process through the spatial clustering of water quality parameters.

### Limitation and Future Research

We developed spatially adaptive machine learning models to predict irrigation water quality in Northern Iran, a region facing significant river pollution challenges. However, this study has some limitations. While long-term water quality data were utilized for model development, the analysis did not explicitly account for seasonal variations in physicochemical parameters. These variations are primarily driven by fluctuations in pollutant loading and river discharge between high-flow and low-flow periods, which exhibit predictable temporal patterns. Incorporating seasonally divided datasets or integrating seasonality as a feature in the modelling process could enhance the model’s ability to capture temporal trends and improve overall predictive performance.

Spatial variables such as geological units, land use type and fragmentation, and population density were not explicitly incorporated as predictive features within each cluster for IWQI modelling. However, certain water quality parameters may be directly influenced by specific land use types or geological conditions. Incorporating high-resolution spatial data could help define more distinct clusters by introducing unique local characteristics, thereby improving pollutant source identification and overall model accuracy. Therefore, future research should address these factors to more comprehensively assess their influence on water quality modelling.

## Conclusion

This study aimed to model the IWQI within a spatially heterogeneous watershed. Considering differences in water quality across the watershed, we identified important water quality parameters for each cluster and applied several ML models, including SVM, RF, ET, XGBoost, DT, and ERT to predict water quality levels. Our results suggest that spatially adaptive clustering may play an important role in improving the accuracy of local prediction models. Considering both water quality data and geographic locations, the studied watershed could be divided into three clusters. Each cluster was linked to a unique set of key parameters for predicting water quality. Among ML models, XGBoost was superior for water quality prediction in all three clusters. The sensitivity analysis demonstrated the importance of key parameters (EC, SAR, Tem_H) affecting IWQI within the three clusters. Accurate predictions of water quality can be attained with locally optimised prediction models using specific sets of parameters (Sect. 3.5) as input combinations. This study offers preliminary guidance for water resource management by proposing locally tailored prediction models based on the IWQI, which were evaluated using water quality data collected from multiple stations over an extended period. These models could support targeted interventions and protection measures. Overall, the approach presented aims to address some of the complexities inherent in predicting water quality across spatially heterogeneous environments and over time. However, future research could further explore the potential of other ML algorithms, such as deep learning or hybrid models, to improve the predictive performance.

## Data Availability

The data sets used in this study are available from the corresponding author upon reasonable request.

## Abbreviations

ML: Machine learning
IWQI: Irrigation Water Quality Index
TDS: Total Dissolve Solid
SOA: Sum of Anions
SOC: Sum of Cations
Tot_H: Total Hardness
Tem_H: Temporary Hardness
KR: Kelly's Ratio
MH: Magnesium Hazards
Na%: Percent Sodium
PI: Permeability Index
SAR: Sodium Absorption Ration
SSP: Soluble Sodium Percentage
RF: Random Forest
BRT: Boosted Regression Tree
XGBoost: Extreme Gradient Boosting
ET: Extra Trees
SVM: Support Vector Machine
DT: Decision Tree
LULC: Land Use and Land cover
